# Applications of speckle tracking echocardiography in paediatric and congenital heart disease: an expert consensus paper from AEPC Imaging Working Group

**DOI:** 10.1093/ehjimp/qyag132

**Published:** 2026-07-27

**Authors:** Massimiliano Cantinotti, Tristan Ramcharan, Dan Mihai Dorobantu, Colin McMahon, Almudena Ortiz Garrido, Margarita Brida, Giulia Pasqualin, Francesca Raimondi, Beatrice Bonnello, Jaroslaw Meyer-Szary, Anna Sabaté Rotés, Owen Miller, Henrik Brun, Misha Bhat, Gabriela Doros, Heynric Grotenhuis, Peter Olejnik, Martin Koestenberger, Andriana Anagnostopoulou, Laurens Koopman, Diala Khraiche, Markku Leskinen, Inga Voges, Jan Sunnegårdh, Sylvia Krupickova, Mari Nieves Velasco, Alexander Van De Bruaene, Giovanni Di Salvo

**Affiliations:** National Research Council, Tuscany Region Foundation G. Monasterio, Massa, Italy; Cardiovascular Clinical Academic Group, City St George's, University of London, Northampton Square, London EC1V 0HB, UK; Heart Unit, Birmingham Children's Hospital, Steelhouse Lane, Birmingham B4 6NH, UK; Children’s Health and Exercise Research Centre, University of Exeter, Exeter, UK; Cardiology Department, Bristol Heart Institute, Bristol NHS Trust, Bristol, UK; Department of Paediatric Cardiology, Children's Health Ireland at Crumlin, Dublin, Ireland; Department Pediatric Cardiology, Hospital Materno-Infantil, Malaga, Spain; Department of Medical Rehabilitation, Medical Faculty, University of Rijeka, Rijeka, Croatia; Pediatric Cardiology and Adult Congenital Disease Heart Centre, IRCCS Policlinico San Donato, San Donato Milanese, Milan, Italy; Division of Pediatric Cardiology, Spedali Riuniti, ASST Azienda Ospedaliera Papa Giovanni XXIII, Bergamo, Italy; Department of Paediatric Cardiology, Great Ormond Street Hospital, London, UK; Department of Paediatric Cardiology and Congenital Heart Defects, Faculty of Medicine, Medical University of Gdańsk, Gdańsk, Poland; Cardiologia Pediatrica, Hospital Universitari Vall d'Hebron, Barcelona, Spain; Department of Paediatric Cardiology, Evelina Children Hospital, London, UK; Department of Pediatric Cardiology, Oslo University Hospital, Oslo, Norway; Department of Pediatric, Skane University Hospital and Lund University, Lund, Sweden; Victor Babes University of Medicine and Pharmacy Timisoara, Louis Turcanu Emergency Hospital for Children, Timisoara, Romania; Department of Paediatric Cardiology, University Medical Centre Groningen, Groningen, The Netherlands; Department of Pediatric, Comenius University, Bratislava, Slovakia; Division of Paediatric Cardiology, Department of Pediatrics, Medical University, Gratz, Austria; Department of Cardiology, Aghia Sophia Children Hospital, Athina, Greece; Division of Cardiology, Department of Paediatrics, Erasmus MC Sophia, Rotterdam, The Netherlands; Department of Pediatric, Necker Hospital, Paris, France; Paediatric Cardiology, Oulu University Hospital, Oulu, Finland; Department for Congenital Cardiology and Pediatric Cardiology, University Hospital Schleswig-Holstein, Campus Kiel, Kiel, Germany; Children’s Heart Center, The Queen Silvia Children´s Hospital, Gothenburg, Sweden; Department of Paediatric Cardiology, Royal Brompton, London, UK; Department of Paediatric Cardiology, Bristol Royal Hospital for Children, Bristol, UK; Department of Cardiology, University Hospitals Leuven, Lueven, Belgium; Paediatric Cardiology and Congenital Heart Disease, Woman and Children’s Health Department, University of Padua, Experimental Cardiology, Paediatric Research Institute (IRP), Padua, Italy

**Keywords:** echocardiography, children, congenital heart disease, speckle tracking echocardiography, ventricular function, atrial function, ventricular strain, atrial strain

## Abstract

This document has been developed to provide a practical guide for the application of speckle tracking echocardiography (STE) in paediatric and congenital heart diseases. In the present document, the following topics are reviewed and discussed: (i) the basic principles of STE, its potential applications, and limitations in the paediatric population; (ii) normal paediatric reference values for STE; and (iii) the use of STE in various congenital and acquired heart diseases.

The potential role of routine STE in the management and follow-up of paediatric and congenital heart disease is explored, with practical examples illustrating how STE can provide additional insights beyond conventional echocardiographic parameters. Limitations regarding clinical interpretation, prognostic value, the lack of standardized timing for measurements, and the absence of universally accepted thresholds for STE-derived strain parameters are also highlighted.

Overall, this document is intended to serve as a comprehensive guide to (i) orient clinicians regarding the strengths and limitations of STE in paediatric and congenital heart disease; (ii) identify specific clinical scenarios where STE may be particularly informative; and (iii) encourage future research and multicentre collaborative studies. By providing both practical guidance and a framework for further investigation, the document aims to support standardized use of STE, improve patient care, and foster the integration of this technique into routine paediatric and congenital cardiology practice.

## Background

Speckle tracking echocardiography (STE) has emerged as a reliable diagnostic tool for the early detection of ventricular systolic dysfunction.^1,2^ While its applications are well established in adult cardiology,^1,2^ as highlighted in recent guidelines,^1,2^ the use of STE in paediatric cardiology has thus far remained more limited. Nevertheless, increasing evidence supports its role in children with both congenital heart disease (CHD) and acquired heart disease,^3–6^ as well as in adults with CHD.^3–6^

In paediatric populations, ventricular STE has been particularly applied in the longitudinal follow-up of specific CHDs after surgical correction or palliative procedures,^3–6^ including tetralogy of Fallot (TOF),^7–19^ univentricular heart following staged Fontan palliation,^2,5,20–39^ transposition of the great arteries (TGA) after arterial switch operation,^3,33,39–68^ anomalous origin of the left coronary artery from the pulmonary artery (ALCAPA),^69–72^ aortic stenosis,^3,73,74^ and aortic coarctation.^75–80^ STE-based assessment of right ventricular function has also proven valuable in children with pulmonary hypertension of various aetiologies.^81–97^

Moreover, STE has been investigated for its potential in detecting early ventricular dysfunction following paediatric cardiac surgery^52,98–105^ and in the longitudinal monitoring of heart transplant recipients.^25,106–114^ In paediatric electrophysiology, STE has been used to assess ventricular function and dyssynchrony in children with heart block and other arrhythmias.^115–128^

Beyond structural and rhythm-related conditions, ventricular STE has been widely adopted for the detection of subclinical ventricular dysfunction in children with cardiomyopathy,^6,129–135^ cardiotoxicity in paediatric oncology patients,^136–149^ as well as for the evaluation of ventricular involvement in inflammatory cardiac diseases (such as myocarditis, Kawasaki disease and multisystem inflammatory syndrome in children (MIS-C) related to COVID-19).^150–163^ In addition, atrial strain analysis has gained increasing recognition as a complementary parameter for the assessment of diastolic dysfunction in CHDs, including TGA,^67,68^ TOF,^17–19^ univentricular heart,^25,36–39^ and pulmonary hypertension.^95–97^

In recent years, advances in cardiovascular imaging and the increasing emphasis on precision phenotyping have expanded the role of STE in congenital and paediatric cardiology.^3–6^ However, important challenges remain in these populations, including complex cardiac anatomy, age-related physiological variability, and limited standardization across vendors and software platforms.^3–6^ Although previous reviews have summarized technical aspects and disease-specific applications of STE, a comprehensive synthesis specifically focused on paediatric and CHD populations, integrating current evidence with practical considerations and emerging clinical applications, remains timely and necessary. Given the rapid expansion of STE use in congenital cardiology, a dedicated paediatric/CHD-focused framework is increasingly needed to support more standardized implementation, interpretation, and future research directions.

The aims of this manuscript are to:

1. demonstrate feasibility of STE in paediatric cardiology in different clinical settings;
2. show current evidence on the diagnostic and clinical utility of the use of speckle tracking in paediatric echocardiography and in adults with congenital heart disease;
3. indicate when STE should be routinely used as an integral part of diagnosis, and when it may be considered an additional diagnostic tool;
4. provide a guide for easy, fast, and reproducible use of STE in paediatric echocardiography and in adults with congenital heart disease;
5. provide a guide for development of basic training, teaching, quality improvement, and research programmes.

Objectives

1. Define feasibility of STE analysis in paediatric cardiology
2. Identify current evidence to support the use of STE in paediatric echocardiography and in adults with congenital heart disease.
3. Define when STE should be routinely used and when it may be used as an additional diagnostic tool.
4. Define strategies for easy, fast, reproducible, and affordable use of STE in paediatric echocardiography and in adults with congenital heart disease.
5. Define strategies for optimizing training, quality improvement, and research.

This document was developed in collaboration with the Imaging Working Group of the Association for European Paediatric and Congenital Cardiology and the Congenital Heart Disease Shared Interest Group (SIG) of the European Association of Cardiovascular Imaging.

The document follows criteria for the expert consensus paper of the ESC.^164^ Categories indicated in the ESC Clinical Consensus Statement have been used for clinical advice.^165^

### STE basic principles, advantages, and limitations in the use in the paediatric population

#### Basic principles

STE is an advanced echocardiographic technique that allows quantitative assessment of myocardial deformation by tracking natural acoustic markers (‘speckles’) within the myocardium throughout the cardiac cycle. Unlike Doppler-based methods, STE is angle-independent and provides multidirectional strain analysis, including longitudinal, circumferential, radial, and area strain. This enables a more sensitive evaluation of systolic and diastolic function, often detecting subclinical myocardial dysfunction before alterations in conventional echocardiographic parameters such as ejection fraction become apparent.

#### Advantages

In the paediatric setting, STE offers unique opportunities. It has the potential to (i) detect early ventricular dysfunction in children with congenital or acquired heart disease, even when traditional measures remain normal; (ii) provide robust functional follow-up in surgically palliated or corrected congenital heart defects (e.g. tetralogy of Fallot, Fontan circulation, transposition of the great arteries); (iii) monitor cardiotoxicity in paediatric oncology patients, offering early markers of chemotherapy-induced myocardial impairment; (iv) assess ventricular and atrial function in systemic or pulmonary hypertension, inflammatory conditions (e.g. myocarditis, Kawasaki disease, MIS-C), and post-transplant settings; and (v) contribute to the evaluation of electromechanical dyssynchrony in paediatric arrhythmias. Furthermore, atrial strain analysis is emerging as a promising complementary tool to assess ventricular diastolic function and atrial contribution to cardiac filling in congenital and acquired diseases.

#### Limitations

Despite its clear advantages, several limitations affect the application of STE in paediatric populations. Normative data remains limited and heterogeneous, with substantial variability related to vendor-specific platforms, software versions, tracking algorithms, and imaging protocols.^1–4^ Inter-vendor reproducibility remains a major challenge, as strain values derived from different software packages may not be directly interchangeable, limiting comparison across studies and reducing the applicability of universal cut-off values. Although ongoing standardization initiatives have attempted to improve consistency, complete harmonization across platforms has not yet been achieved.^1–4^

Paediatric-specific factors further complicate STE acquisition and interpretation. Reproducibility may be affected by elevated and age-dependent heart rates, particularly in infants and neonates, where accurate temporal resolution requires optimization of frame rates and may still result in reduced tracking performance.^1–4^ The small cardiac size and rapid myocardial motion characteristic of younger children increase susceptibility to tracking errors and may reduce measurement precision. In addition, image acquisition frequently depends on patient cooperation.^1–4^ Motion artefacts, inability to maintain breath-holding, and limited cooperation in infants and younger children may compromise image quality, while sedation, although occasionally required, can alter loading conditions and heart rate, potentially influencing strain measurements.^1–4^

Technical feasibility may also be reduced in patients with complex congenital anatomy or postoperative conditions. Abnormal ventricular geometry, heavy trabeculations, surgically altered anatomy, patch material, and thoracic deformities may impair endocardial border delineation and tracking accuracy.^1–4^ Similarly, suboptimal acoustic windows may occur in overweight patients or after multiple surgical procedures. These challenges are particularly relevant in complex CHD populations, where strain analysis may be technically demanding and interpretation less straightforward.^1–4^

Inter-study comparability is further restricted by the absence of universally standardized acquisition and analysis protocols, including differences in image acquisition settings, contour tracing approaches, and selection of cardiac cycles for analysis.^1–4^ Finally, STE requires dedicated training and technical expertise, and visual verification of tracking quality remains essential, which may limit its widespread implementation in routine paediatric echocardiography.^1–4^

## Normal values

Over the past decade, multiple nomograms for paediatric left ventricular (LV) strain and strain rate have been developed using STE.^2,166–203^ As with other echocardiographic functional parameters, correlations of STE-derived strain values with anthropometric indices [e.g. body surface area (BSA), weight, height] and chronological age have generally been weak. Consequently, most available studies have opted for age-based normalization^166–169,171,173,176–189,192^ and reported data as mean ± standard deviation.^172–174,177–187,189,192^ Although heart rate was routinely measured, formal heart rate normalization was applied only in one study.^183^ Another group proposed z-score equations indexed to BSA; however, the correlations between strain parameters and BSA were extremely weak (*R*^2^ ranging from 0.03 to 0.002), introducing potential bias into these equations.

Methodological refinements have been proposed to overcome such limitations. Three-dimensional echocardiography (3DE) offers potential advantages, including more comprehensive volumetric and spatial assessment and avoidance of speckle loss due to out-of-plane motion.^173^ More recent multicentre and larger-sample studies have employed standardized protocols, thus improving reproducibility and comparability.^181,192^ Nonetheless, intrinsic factors—such as elevated heart rates, smaller chamber dimensions, and rapid maturational changes—make strain measurements particularly unreliable in neonates and young infants.^193^ Beyond the first year of life, strain values exhibit greater stability and reproducibility across age groups.^194^ For example, reported LV global longitudinal strain (GLS) in a 3-year-old child ranged from −23.6%^179^ to −20.7%^177,178^ and −21.3%,^195^ whereas in a 14-year-old the range was narrower, from −21.8%^177–189^ to −22.7%.^183^

Two recent meta-analyses proposed using a single reference range for paediatric LV and right ventricular (RV) strain,^175,182–193^ based on the relatively small variation across age. However, such an approach oversimplifies the underlying maturational trends and is inconsistent with the fundamental principle of nomogram-based interpretation.^21,27^ Adequately stratified large-scale datasets remain necessary to fully define the age-related trajectories of strain parameters.

Data on rotational mechanics—including basal–apical rotation, torsion, twist, and untwist—remain sparse in paediatric cohorts.^195–197^ In a 2DSTE study of 80 children aged 3 months to 15 years, one research group found that apical rotation, basal rotation, and twist (normalized to LV length) decreased progressively with age, whereas untwisting recoil showed no significant age dependency. Another study demonstrated in a broader age spectrum (3–40 years) that twist, untwist, and deformation rates were highest in the youngest individuals, with stronger correlations to heart rate than to chronological age. A large 3DSTE series including 311 subjects from childhood to adulthood showed that torsion values were lowest in children/adolescents and progressively increased to reach a plateau in adulthood.^199^

### Ventricular strain

#### Relationship with age and body size

Several investigations have documented mild, inverse correlations between LV strain parameters and age, with correlation coefficients ranging from −0.8 to −0.007.^184,185^ Importantly, these relationships are non-linear, as confirmed by large paediatric datasets.^184,185^ LV GLS values are highest in infants and toddlers (31 days–24 months), while adolescents (11–18 years) exhibit significantly lower values.^185,191^ In contrast, LV circumferential strain (CS) varies minimally with age and is slightly higher in older children.^30^ In a cohort exceeding 1000 subjects under 21 years, both LV longitudinal and circumferential strain peaked at approximately 4–5 years of age before declining;^192^ RV longitudinal strain followed a similar trend.^192^ Weak inverse correlations with BSA have also been described.^192^

#### Sex differences

Most studies have failed to demonstrate clinically relevant sex-based differences.^195^ When present, differences are minimal, typically confined to adolescence and possibly attributable to variations in pubertal maturation.^185,191^ Some series reported slightly higher LV and RV strain in females, but findings were inconsistent across studies.^183^

#### Inter-vendor variability

Vendor-related variability is a recognized limitation in STE.^194,198,199^ Agreement between GE and TomTec platforms is generally good for LV GLS (ICC 0.88–0.90) but less satisfactory for LV CS, particularly in children under 3 years.^194,198,199^ Discrepancies are more pronounced in comparisons between Philips QLAB (versions 10.5, 10.8, AutoSTRAIN) and TomTec, with older QLAB versions yielding the greatest divergence.^194,198,199^ Agreement between GE and Philips platforms is poor, with ICCs as low as 0.34 for LV GLS and 0.12 for circumferential strain. Intra-vendor differences also exist: QLAB 10.5 tends to produce higher strain values than QLAB 10.8 and AutoSTRAIN, especially in older children.^194,198,199^

#### Inter- and intra-observer variability

Reproducibility depends on both software platform and frame rate. For Philips QLAB 10.8, intra-observer ICC for LV strain is moderate (0.50–0.79), while inter-observer ICC ranges from mild to good (0.80–0.96).^185,191,194,198,199^ AutoSTRAIN provides excellent intra-/inter-observer reproducibility, particularly for LV GLS.^194,198,199^ TomTec and GE software similarly show good reproducibility, with ICCs >0.85 for LV GLS and slightly lower values for CS. Higher frame rates consistently improve reproducibility.^194,198,199^

### Atrial strain

#### Maturational variation

Atrial strain parameters, including left atrial (LA) reservoir strain (LASr), conduit strain, and contractile strain, display significant age-related changes.^168,171,184–187^ 2DSTE-based nomograms demonstrate a nonlinear positive correlation between LASr and age, and a nonlinear negative correlation between LA contractile strain and age, with the most rapid changes during infancy.^168,171,184–187^ Both LA and right atrial (RA) conduit strain values are lower in younger children, while contractile strain values are higher during early life.^168,171,184–187^ Using 3DSTE, all LA strain components decline with age, although correlations are weak (*r* = 0.14 for global GLS; *r* = 0.31 for global 3D strain).^186^

#### P-gating vs. R-gating and software differences

Across all paediatric age groups, P-wave-gated measurements yield consistently lower strain values than R-wave-gated measurements for both LA and RA.^184,195^ Marked differences also exist between atrial-specific software (QLAB 10) and older ventricular-specific software adapted for atrial analysis (QLAB 9).^184,194^ LASr values are systematically lower with atrial-specific software across all ages (*P* < 0.001), while RA Sr values differ significantly only in certain age groups. LA conduit strain (LASct) values tend to be higher in younger children and lower in older children when assessed with QLAB 10 compared to QLAB 9.^184,185,195^

*Tables 1* and *2* summarize the principal paediatric echocardiographic nomograms for STE-derived ventricular and atrial strain parameters.

**Table 1:** Major paediatric STE nomograms for ventricular strain values

Author	Population	Measures	Software	Data norm	Data expression
Adar A 2019 USA^166^	*N* = 312 3 days–20.5 years	LV LS, CS and synchrony;	Echo: Philips Epiq Software: QLAB v0.10.5 (Philips)	Age groups	Mean, SD
Harrington JK 2021 USA^168^	*N* = 577 1–18 years	LV SR	Echo: Philips Software: QLAB (Philips)	Age	Z-scores
Koopman LP 2019 The Netherlands^171^	*N* = 103 mean 10.8 years IQR 7.3–14.3 years.	LV LS, CS	Echo: Philips IE33 Software: QLAB 9.0 (Philips)	Age groups	Mean, SD Percentiles
Romanowicz J 2023 USA^191^	*N* = 1032 < 21 years old	LV and RV LS, LV CS	Echo: Philips Epiq Software: Auto-strain, QLab 10.5 10.8	Age	Mean, SD Z-scores
Kamel H 2022 Egypt^173^	*N* = 200 3.832+/−1.522 years range 0.1–5.9 years	LV GLS, GCS, GRS 2d and 3D	Echo: Vivid E9 (GE). Software: EchoPAC V113 (GE).	Age groups	Mean, SD
Kotby AA 2023, Egypt^169^	*N* = 250 1–16 years	LV LS	Echo: GE Software: EchoPAC	Age groups	Mean, SD
Aristizábal-Duque CH 2022 Spain^174^	*N* = 156 6 t- 17 years	LVGLS, RVGLS, RV free wall LS, LA	Echo: Philips IE33 Software: 13.0 of QLab 13 (Philips)°	Age groups, BSA	Mean, SD
Klistisic L, 2013 The Netherlands^178^	*N* = 183 0–19 years	LV LS, CS, RS	Echo: Vivid 7 GE Software: EchoPAC GE	Age groups	Mean, SD
Marcus K, 2011 USA ^177^	*N* = 144 0–19 years	LV LS, CS, RS	Echo: Vivid 7 GE Software: EchoPAC GE	Age groups	Mean, SD
Zhang L, 2013 China^172^	*N* = 226 0–18 years	LV 3D STE LS, CS, RS	Echo: Philips IE33 Software: Tomtec	Age groups	Mean, SD
Cantinotti M, 2018 Italy ^180^	*N* = 721 31 days-18 years	LV, LS, CS RV LS	Echo: Epiq/IE33 (Philips) Software: Q-LAB 9 Philips	Age groups, gender	Mean, SD
Dallaire F, 2016 Canada^179^	*N* = 233 1–18 years	LV LS, CS	Echo: Vivid 7 GE Software: EchoPAC GE 7	BSA	Z-scores
Acheampong B, 2023 USA^163^	*N* 142 0–18 years	LV LS, CS, RS	Echo: Philips and Siemens Software: Cardiac Performance Analysis version 3.0*	Age groups	Percentiles

CS, circumferential strain; GCS, global circumferential strain; IQR, interquartile range; LA, left atrium; LV, left ventricle; LS, longitudinal strain; N, number; GLS, global longitudinal strain; GRS, global radial strain; RV, right ventricle; RS, radial strain; SD, standard deviation; SR, strain rate; GE, General Electric Ultrasound, Horten, Norway, EPIQ platform, Philips Healthcare, Andover, Massachusetts; TomTec, TomTec Imaging Systems, Germany, Siemens Healthineers Erlangen, Germany °which integrates the TOMTEC auto-strain software; *Independent software.

**Table 2:** Major paediatric STE nomograms for atrial strain values

Author	Population	Measures	Software	Data norm	Data expression
Cantinotti M, 2019, Italy^180,184^	*N* = 836 31 days–18 years	2D LA and RA strain	Echo: Epiq/IE33 (Philips) Software: Q-LAB), and Q- LAB 10° (Philips)	Age groups	Mean, SD
Ghelani S, 2013 USA^185^	*N* = 196 4 days–20.9 years	3D LA volumes and strain	Echo: Philips IE33 Software: 4D LV Analysis, Tomtec 3.1	Age	Z-scores
Kutty S 2013, USA^186^	*N* = 153 3–20 years	2D LA and RA strain	Echo: GE Vivid 7, Software: EchoPAC Bt11 GE	Age groups	Mean, SD
Jimbo S 2020 Japan^167^	*N* = 112 (median 12.0 years; range 6–16 years)	2D LA strain and SR	Echo: NR Software: TomTec	Age groups	z-scores
Aristizábal-Duque CH 2022 Spain^174^	*N* = 156 6 t- 17 years	2D LA strain	Echo: Philips IE33 Software: 13.0 of QLAB 13 (Philips)°	Age groups, BSA	Mean, SD

LA, left atrium; *N*, number; NR, not reported; RA, right atrium; SD, standard deviation; SR, strain rate; 2D, two-dimensional; 3D, three dimensional; SD, standard deviation° dedicated to atria. Epiq, iE33 systems (Philips Medical Systems, Bothell, WA, USA), QLAB 9; Philips Medical Systems, Andover, MA, USA), (QLAB 10; Philips Medical Systems, Andover, MA, USA, Tomtec Imaging System Freisinger Str. 9, 85716 Unterschleissheim, Germany, Vivid E 9/95 GE = General Electric Health Care, Chicago, Illinois, USA.

##### Clinical advice

**Figure 1:**
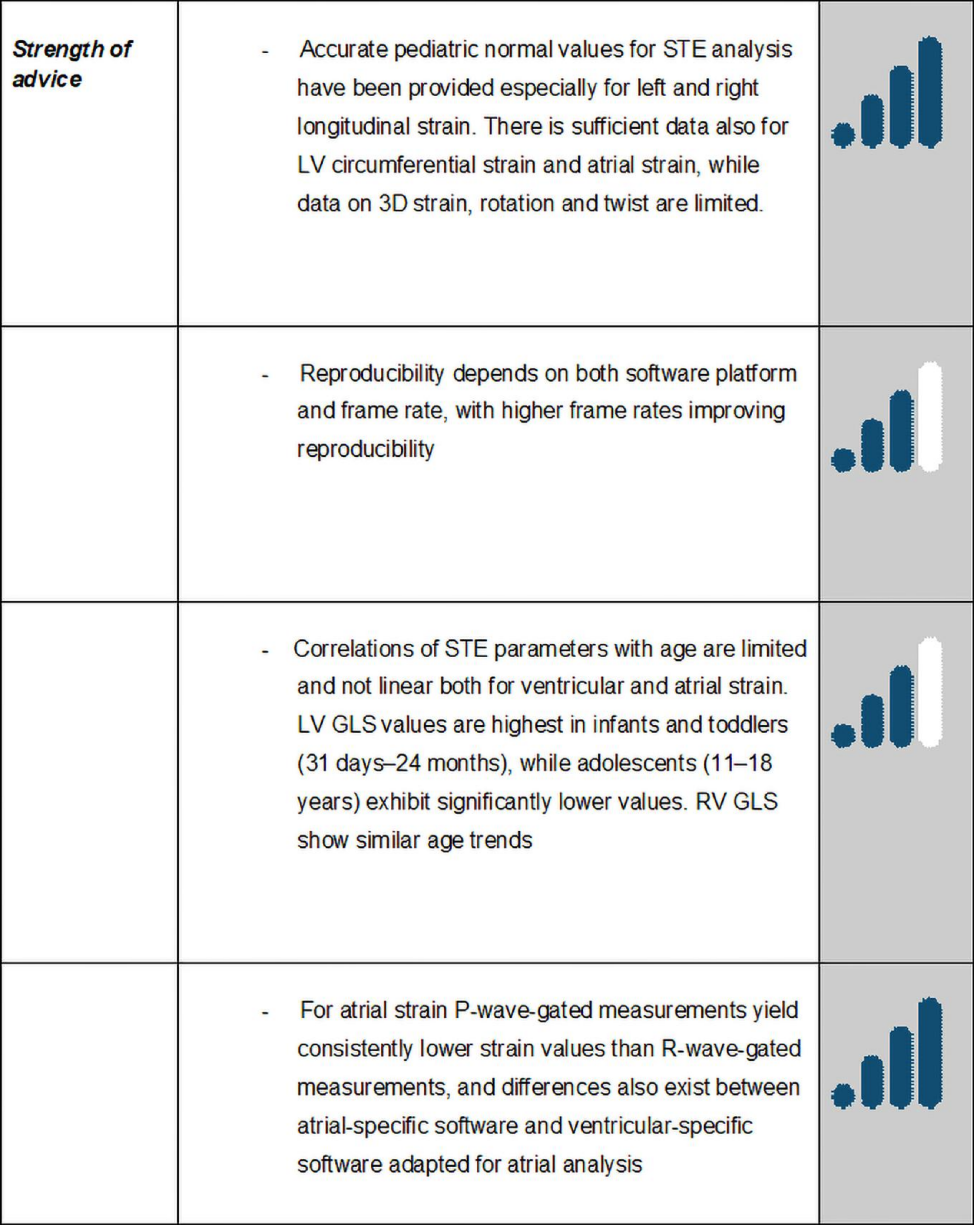
Clinical advice on accuracy and limitation of ventricular and atrial STE normal values, and intervendor variability.

**Gaps in knowledge:** (*Figure 1*) intervendor differences and infra-vendor reproducibility need to be standardized yet.

### STE applications in different congenital and acquired heart diseases

#### Septal defects

STE has increasingly been applied in patients with septal defects to detect subtle myocardial and atrial functional abnormalities that may not be identified by conventional echocardiographic parameters. In atrial septal defect (ASD), STE has shown utility in evaluating biatrial remodelling and monitoring functional recovery after defect closure. Early studies demonstrated persistent alterations in atrial deformation after both surgical and percutaneous ASD closure,^204–207^ suggesting that functional remodelling may continue despite anatomical correction.^204^ In a study by Hajizeinali *et al*.^205^ including 64 patients after surgical or device ASD closure, both right and left atrial strain parameters remained altered at midterm follow-up, highlighting ongoing atrial dysfunction despite successful intervention. Beyond functional assessment, STE-derived atrial parameters may also provide prognostic information. In a prospective study of 73 secundum ASD patients, Vitarelli *et al*. showed that reduced right atrial peak strain and increased atrial mechanical dyssynchrony were associated with subsequent paroxysmal atrial fibrillation development; specifically, right atrial time-to-peak strain dyssynchrony independently predicted arrhythmic risk (*P* = 0.023).^208,209^ Data for ventricular septal defect (VSD), are limited. In 60 children with VSD significantly impaired global longitudinal strain were found together with elevated cardiac troponin-I levels compared with controls (*P* < 0.001), suggesting early subclinical myocardial dysfunction related to chronic volume overload.^210^

##### Clinical advice

**Figure 2:**
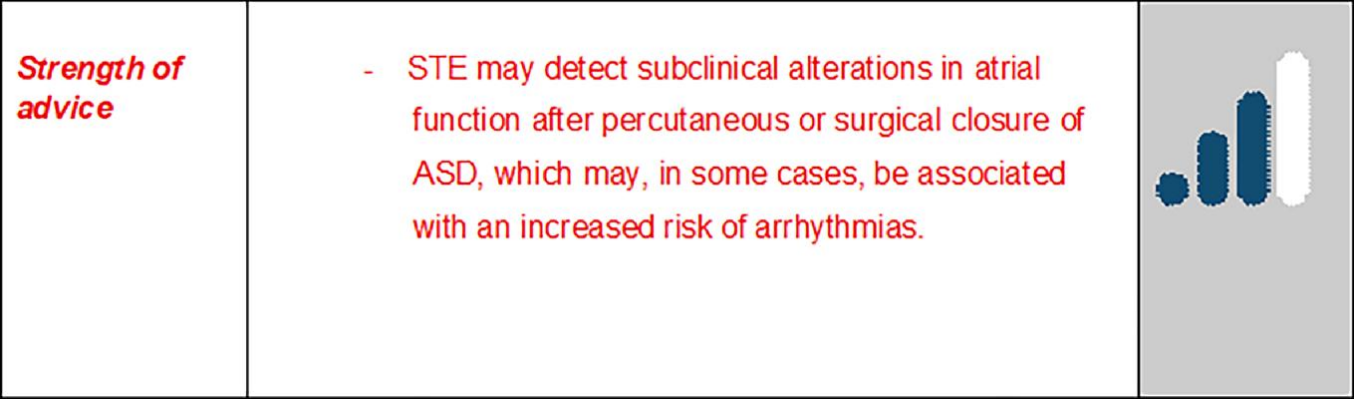
Clinical advice on application of STE in septal defects.

**Gaps in knowledge:** (*Figure 2*) The clinical and prognostic role of STE in predicting long-term survival, arrhythmogenic risk, and associations with cardiorespiratory function in ASD after surgical/percutaneous closure need to be defined yet.

#### Transposition of the great arteries after arterial switch operation

STE has emerged as a sensitive tool for detecting subclinical myocardial dysfunction in patients with transposition of the great arteries (TGA), providing complementary information to conventional echocardiographic indices such as ejection fraction (EF) and fractional shortening (FS).^40–48^ In adolescents and young adults after arterial switch operation (ASO), studies in 53 patients (mean age 16 ± 3 years) demonstrated significantly increased LV stiffness and reduced global GLS (GLS, −17.4 ± 2.6%) compared with age-matched controls (−19.5 ± 2.1%, *P* < 0.001), despite preserved LV EF (61 ± 4%).^40^ GLS correlated with LV stiffness index (*r* = −0.52, *P* < 0.001), suggesting that strain abnormalities reflect intrinsic myocardial remodelling undetectable by conventional EF.^40^ Other cohorts of 64 ASO survivors (mean age 13.9 ± 4.2 years) confirmed impaired GLS (−17.6 ± 2.3%) and circumferential strain (−20.3 ± 3.1%) despite normal EF (58 ± 5%, *P* < 0.001 vs. controls),^43^ indicating persistent subclinical LV dysfunction, possibly related to altered coronary flow dynamics or surgical manipulation of the root and great arteries.^40,43^

The underlying mechanisms for subclinical LV impairment are not fully elucidated. Proposed contributors include early coronary malperfusion following ASO,^49^ progressive neo-aortic regurgitation, and presence of a gothic arch,^51^ which may reflect abnormal aortic wall properties and correlate with worse LV strain. Factors related to the ASO procedure itself, such as older age at ASO,^52^ longer bypass time, and restrictive PFO at birth,^53^ have also been linked to lower LV and RV strain during follow-up.

In the early postoperative setting, assessments in 54 infants (median age 5 days) showed preoperative GLS (−13.5 ± 2.8%) significantly associated with postoperative length of hospital stay (*r* = 0.43, *P* = 0.02), whereas EF and FS did not predict outcome.^41^ Similarly, preoperative LVLS in neonates with TGA (−14.2 ± 1.8% vs. −18.9 ± 2.1% in controls, *P* < 0.001)^47^ and abnormal LV torsion mechanics in 21 ASO patients with intact ventricular septum (mean age 13 ± 3 years) demonstrated reduced basal rotation (*P* < 0.05) and torsional deformation, despite normal EF.^42^ Other cohorts of 40 paediatric ASO patients (mean age 10 ± 4 years) confirmed lower GLS and strain rate compared with controls (*P* < 0.01), with preserved EF and FS.^46^

STE-derived functional assessment may identify patients at risk of worse outcomes and guide late intervention. Machine-learning clustering combining STE and TDI data revealed subgroups with lower cardiac performance associated with older age at ASO, more reinterventions, higher pulmonary artery gradients, and more frequent neo-aortic regurgitation,^54^ despite normal conventional biventricular function.^50^ However, there are no reports on STE predicting mortality or major adverse cardiac events in this population, likely due to young age and low event rates.^3^ LV strain at rest post-ASO was not correlated with cardiorespiratory fitness measured by exercise testing, possibly due to maintained cardiac reserve early in life, and stress echocardiography data remain limited.^49,55^

The impact of atrial and ventricular coupling abnormalities has been more recently evaluated using atrial strain imaging.^67,68^ In a cohort of 35 adults after ASO, LA reservoir strain was significantly reduced (24.5 ± 5.6% vs. 34.7 ± 6.1% in controls, *P* < 0.001), correlating with reduced LV GLS (*r* = 0.56, *P* < 0.001).^67^ LV GLS has also been proposed as a ‘functional fingerprint’ integrating surgical history and long-term myocardial adaptation in ASO survivors.^48^

##### Clinical advice

**Figure 3:**
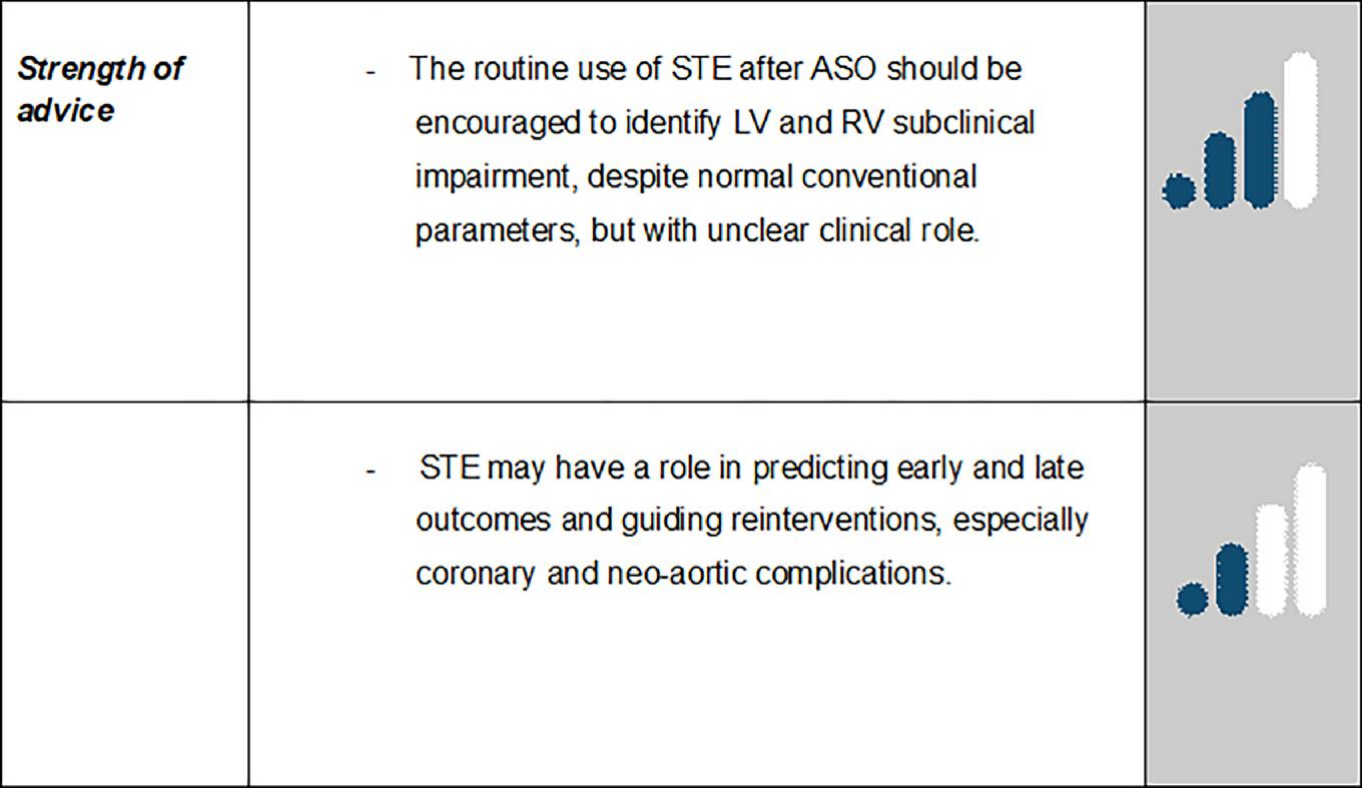
Clinical advice on application of STE in transposition of the great arteries after arterial switch operation.

**Gaps in knowledge (TGA ASO):** (*Figure 3*) The clinical and prognostic STE role in predicting long term survival, late risk stratification and associations with cardiorespiratory function after ASO need to be clarified. Role of initial ASO procedure in late LV and RV impairment needs to be clarified to guide changes to first intervention (especially age at ASO).

#### The systemic right ventricle

In patients after atrial switch for TGA or those with CCTGA, systemic right ventricular (RV) strain measured by STE is a prognostic marker, with a 1% decrease in strain being associated with a 10% increase in risk for major cardiac adverse events or death.^55^ In 186 patients followed prospectively, baseline RV longitudinal strain (RV-LS) was −17% ± 4%, with an annual decline of −4%, which also correlated with outcomes.^56^ Risk factors for RV function decline include LV pacing, LV impairment, and arterial hypertension, highlighting the importance of RV–LV interaction in these patients. Biventricular systolic and diastolic performance is known to be reduced after arterial switch, as shown by STE-derived myocardial work.^57^

Despite the contribution of the sub-pulmonic LV to global cardiac impairment, only tricuspid valve replacement (TVR) was associated with reduction in RV dysfunction progression, whereas medical therapy and resynchronization were not. After TVR, RV strain improved, along with oxygen uptake and exercise capacity, emphasizing the role of STE in evaluating treatment response in this population.^58^ Systemic RV strain is also associated with markers of heart failure, such as NT-proBNP, supporting its use in longitudinal follow-up.^59^

Contraction patterns differ between atrial switch systemic RV and CCTGA. In atrial switch TGA, longitudinal and radial function is impaired, with anteroposterior contraction compensating, whereas in CCTGA all three components contribute equally.^60^ This reflects adaptation to pressure overload and suggests that global function assessment should avoid reliance on visual inspection alone.^61^

Segmental and regional mechanics of the systemic RV are challenging to assess. Although QRS duration increases with disease progression, the association between electrical and mechanical dyssynchrony, which could guide resynchronization therapy, is inconsistent. In 51 patients with systemic RV, abnormal myocardial work by STE was observed, but no clear association with QRS duration was found; however, segmental wall abnormalities were present in those with QRS >120 ms.^62,63^

In patients with D-TGA after atrial switch procedures (Mustard or Senning), STE has proven valuable for evaluating systemic RV function, often underestimated by conventional indices.^45^ In 69 adults (mean age 31 ± 7 years), RV GLS (−13.1 ± 3.5%) and circumferential strain (−12.4 ± 3.2%) were markedly reduced despite preserved systemic RV fractional area change (FAC, 36 ± 5%). Lower RV GLS independently predicted adverse clinical events (hazard ratio 1.48 per 1% decrease, *P* = 0.01) and correlated with NYHA class (*r* = 0.61, *P* < 0.001), whereas conventional EF showed weaker associations.^45^ These findings emphasize the incremental diagnostic and prognostic value of STE-derived parameters in lifelong evaluation of TGA patients with systemic LV and systemic RV physiology.

In patients with TGA after atrial switch repair, deformation imaging has shown a characteristic shift from longitudinal shortening to predominant transverse thickening of the systemic RV. In a cohort of 26 AS-TGA patients, transverse (radial) 2D strain was significantly increased compared with controls and demonstrated a strong correlation with exercise capacity (*R* = 0.80; *P* < 0.0001), outperforming traditional functional indices. These findings suggest that transverse strain may serve as a more sensitive marker of systemic RV performance than longitudinal strain in this population and should be considered in routine follow-up.^211^

Non-invasive diastolic function assessment in systemic RV patients is not standardized due to dual AV and VA discordance and challenges in estimating RV filling pressures. In 47 adults with systemic RV,^64^ half had high subaortic RV filling pressures measured invasively, and 83% had dilated left atria. Using E’ velocities, no differences were observed between patients with and without elevated filling pressures; however, STE-derived left atrial reservoir strain, systemic RV GLS, and subpulmonic LV strain were significantly worse in those with high filling pressures (18.0 ± 7.6% vs. 27.9 ± 10.2%, *P* = 0.0009; −13.0 ± 4.4% vs. −17.9 ± 5.0%, *P* = 0.002; −16.8 ± 5.7% vs. −23.0 ± 3.8%, *P* = 0.001). STE also demonstrated diastolic interactions between the RV and LV, reflecting interplay between filling pressures and dyssynchronous contraction.^65^ This highlights the potential utility of STE-based parameters over conventional algorithms in systemic RV assessment.

Exercise ventricular reserve is reduced in patients with systemic RV and may unmask superior vena cava baffle stenosis, supporting its role as an underutilized prognostic and treatment-guiding tool.^33^ In a cohort of children after Senning operation, dobutamine stress RV assessment demonstrated that reduced stress reserve occurs early in life rather than as a late phenomenon.^66^

##### Clinical advice

**Figure 4:**
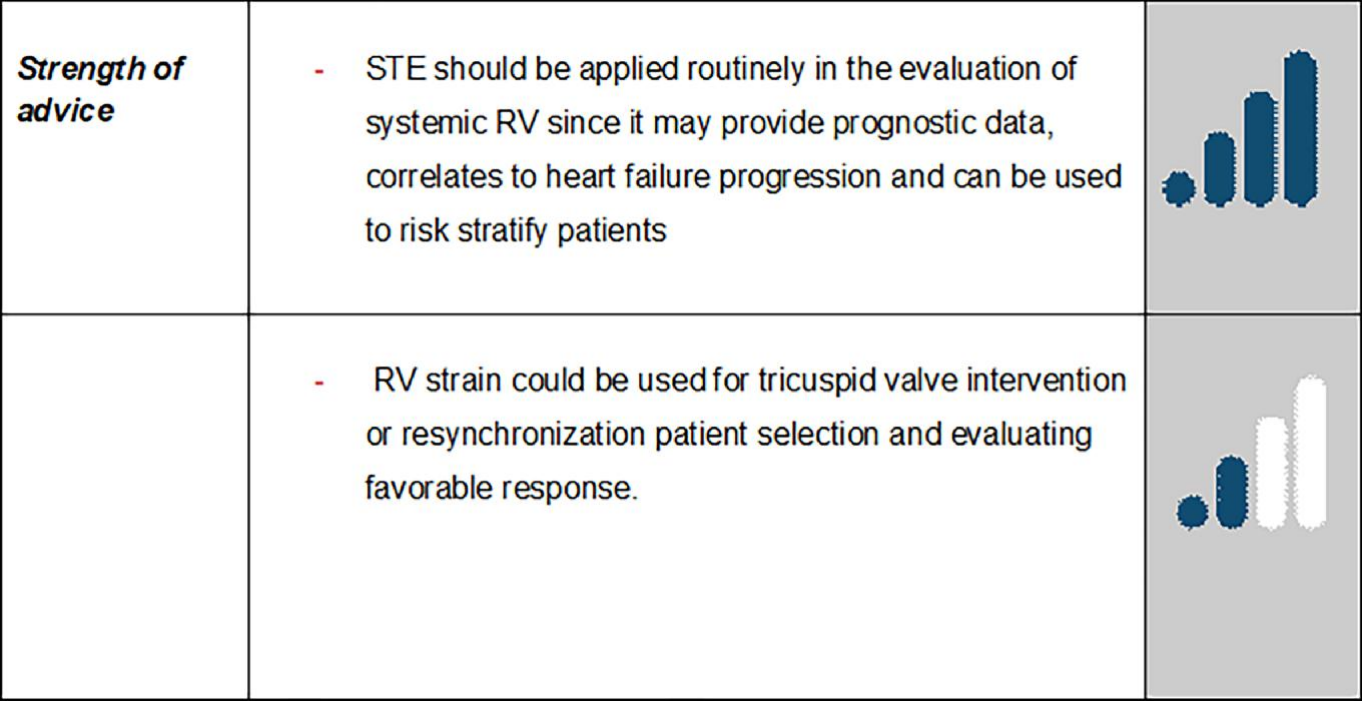
Clinical advice on application of STE in the systemic right ventricle.

**Gaps in knowledge:** (*Figure 4*) Diastolic function of the systemic RV and right-left ventricular interaction play a role in disease progression, but no parameters are yet to be implemented in clinical use.

#### Tetralogy of Fallot

STE has profoundly enhanced the understanding of biventricular mechanics in patients with repaired Tetralogy of Fallot (rTOF), identifying subtle myocardial abnormalities not captured by conventional indices such as EF or FAC.^7–16^ Persistent alterations in both left and right ventricular deformation are now recognized as early markers of adverse remodelling, even in clinically asymptomatic patients.^7–16^

In the LV, studies have consistently documented impaired global and segmental strain despite preserved EF. In one cohort of 60 children and young adults with surgically repaired TOF (mean age 14 ± 6 years), global GLS (GLS) was significantly reduced (−17.5 ± 2.9%) compared with controls (−20.8 ± 2.2%, *P* < 0.001), while EF remained normal (60 ± 5%, *P* = ns).^7^ Segmental analysis indicated the greatest strain reduction in the basal septal and inferior walls, suggesting regional mechanical coupling between surgical scarring and chronic RV overload.^7^ Additional studies in younger cohorts (*n* = 28, mean age 6 ± 3 years) demonstrated intrinsic abnormalities in LV rotational mechanics, with decreased apical rotation (*P* < 0.01) and torsion (*P* < 0.05), independent of RV dilation, supporting the hypothesis of biventricular mechanical interdependence.^11^ In asymptomatic children and adolescents (*n* = 77), LV GLS (−18.2 ± 2.4%) and circumferential strain (−20.6 ± 3.3%) correlated inversely with pulmonary regurgitation severity (*r* = 0.52, *P* < 0.001) and RV end-diastolic volume (*r* = 0.49, *P* = 0.01), indicating load-dependent LV–RV interaction.^10^ Findings from a larger cohort (*n* = 132, median age 16 years) confirmed impaired LV GLS (−18.1 ± 2.2% vs. −20.3 ± 1.8% in controls, *P* < 0.001) and radial strain abnormalities, which correlated with reduced exercise capacity and increased LV stiffness index (*r* = 0.55, *P* < 0.001).^9^

The right ventricle, central to TOF pathophysiology, has also been extensively evaluated using STE.^12,14,16^ In a study of 45 children after TOF repair (mean age 10 ± 3 years), RV free-wall strain was significantly decreased (−18.4 ± 3.1%) compared with controls (−25.2 ± 2.8%, *P* < 0.001), even when RV FAC and tricuspid annular plane systolic excursion (TAPSE) were normal.^16^ Another cohort of 40 rTOF patients revealed heterogeneous regional strain distribution, with mid and apical RV free-wall segments most affected (*P* < 0.001), reflecting localized fibrosis and altered myocardial fibre orientation due to surgical scarring. Reduced RV free-wall longitudinal strain was independently associated with longer QRS duration (*r* = 0.46, *P* < 0.01) and higher pulmonary regurgitant fraction, supporting its role as a sensitive marker of electromechanical remodelling.^14^

These findings may have practical implications for longitudinal management. Reduced RV free-wall strain, particularly when associated with progressive pulmonary regurgitation, QRS prolongation, or declining exercise capacity, may identify patients requiring closer surveillance despite preserved conventional parameters such as FAC or TAPSE.^8,10,14^ In this setting, STE abnormalities should not be interpreted in isolation but integrated with conventional echocardiography and cardiac magnetic resonance (CMR), particularly RV volumetric assessment and pulmonary regurgitant fraction, which remain central for longitudinal risk assessment and timing of pulmonary valve replacement.^8,10^ STE may therefore contribute as an adjunctive marker of early functional deterioration before overt structural changes become evident.^8–10^

Early postoperative evaluation (*n* = 35, median postoperative day 7) demonstrated acute biventricular impairment, with both LV and RV GLS markedly decreased (*P* < 0.001) despite preserved EF and FAC, highlighting the immediate sensitivity of STE after repair.^12^

Regional and global strain abnormalities also evolve over time and may be modulated by pacing or residual haemodynamic lesions. In 27 children with permanent RV pacing after TOF repair followed over 5 years, progressive deterioration in both LV and RV longitudinal strain was observed (*P* < 0.001), despite stable EF and FAC, underscoring the vulnerability of ventricular deformation to electrical dyssynchrony.^13^ Integrative reviews have emphasized the prognostic value of STE, identifying LV and RV strain—particularly RV free-wall and LV GLS—as the most reproducible and clinically relevant deformation parameters for risk stratification, correlating with pulmonary regurgitation, exercise intolerance, and arrhythmia risk.^8^ Although universally accepted intervention thresholds are currently lacking, serial deterioration in strain values may provide incremental information during follow-up and may prompt earlier multimodality assessment, particularly when discordance exists between symptoms and conventional echocardiographic findings.

Beyond ventricular performance, atrial strain imaging has provided insight into atrioventricular interaction and diastolic function.^17–19^ Systematic reviews of multiple studies reported consistent reductions in both LA and RA reservoir strain in rTOF patients compared with controls, correlating with ventricular longitudinal strain and diastolic indices.^17^ In 69 adolescents and young adults after TOF repair (mean age 17 ± 5 years), reduced LA reservoir strain (26.1 ± 7.3% vs. 37.5 ± 6.8% in controls, *P* < 0.001) was associated with lower LV GLS (*r* = 0.61, *P* < 0.001), increased native T1 times, and reduced exercise capacity (*P* < 0.01).^18^ Biventricular and biatrial STE in 44 rTOF patients demonstrated impaired atrioventricular coupling (*P* < 0.001), confirming that mechanical dissynchrony extends beyond the ventricles.^19^

In summary, STE has become a cornerstone in the functional assessment of repaired TOF, allowing quantification of global and regional myocardial deformation in both ventricles^7–16^ and atria,^17–19^ providing diagnostic and prognostic information beyond conventional indices and enabling early detection of subclinical dysfunction and characterization of electromechanical remodelling.^7–19^ Importantly, STE findings may complement standard echocardiography and CMR during longitudinal follow-up, supporting individualized surveillance strategies and potentially identifying patients who warrant closer monitoring before conventional indicators of deterioration emerge.

##### Clinical advice

**Figure 5:**
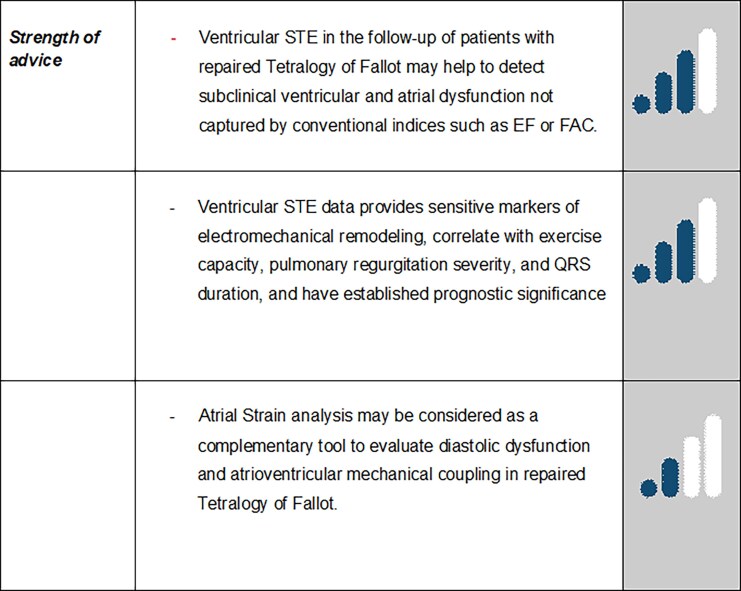
Clinical advice on application of STE in tetralogy of Fallot.

**Gap in knowledge:** (*Figure 5*) The long-term clinical and prognostic relevance, and evolution of ventricular and atrial strain abnormalities in repaired Tetralogy of Fallot remain only partly understood, especially concerning how they relate to electrical dyssynchrony, residual haemodynamic lesions, and progressive myocardial remodelling.

#### Left sided obstruction (aortic stenosis and coarctation of the aorta)

STE has emerged as an essential tool for characterizing myocardial mechanics in left-sided obstructive lesions, including congenital aortic stenosis (AS)^3,73,74^ and coarctation of the aorta (CoA),^75–80^ offering higher sensitivity than conventional systolic indices such as EF and fractional shortening (FS). In congenital AS, deformation analysis demonstrates that LV dysfunction often precedes overt changes in EF.^3,73,74^ In 41 children after balloon valvuloplasty for AS (mean age 7.6 ± 5.1 years), global GLS (GLS) was significantly reduced (−17.2 ± 2.5%) compared with controls (−20.1 ± 2.3%, *P* < 0.001), despite normal EF (61 ± 5%). GLS correlated strongly with peak aortic gradient (*r* = −0.62, *P* < 0.01), highlighting its load-dependent yet clinically meaningful nature.^73^ In 24 infants evaluated before and after balloon aortic valvuloplasty (median age 3 days), LV strain normalized immediately post-intervention (from −14.3 ± 2.1% to −18.7 ± 2.3%, *P* < 0.001) while EF remained unchanged (59 ± 6% vs. 61 ± 5%, *P* = ns), demonstrating the ability of STE to detect rapid recovery of myocardial deformation.^74^ Meta-analytic evidence from 25 studies including 1942 patients confirmed LV GLS as a strong predictor of mortality and morbidity (pooled hazard ratio 1.45 per 1% decrease, *P* < 0.001), providing incremental prognostic value over EF and ventricular volumes.^3^

In CoA, STE has proven valuable across developmental stages. In 92 foetuses (mean gestational age 34 ± 2 weeks), reduced LV strain and strain rate improved prenatal detection of coarctation on the final examination before delivery (sensitivity 87%, specificity 82%), outperforming standard Doppler and dimensional markers.^75^ In 28 neonates with CoA, LV GLS was significantly decreased (−14.8 ± 3.2%) compared with healthy newborns (−19.6 ± 2.7%, *P* < 0.001), despite normal EF and FS, indicating that deformation abnormalities precede geometric remodelling.^76^ In adults with repaired CoA (*n* = 52, mean age 33 ± 11 years), LV GLS (−17.0 ± 2.4%) and circumferential strain (−20.6 ± 3.5%) were reduced compared with controls (*P* < 0.001), despite preserved EF (62 ± 4%), and correlated with residual arch obstruction (*r* = 0.58, *P* < 0.01) and elevated central systolic pressure.^77^ Persistent reductions in GLS (−18.1 ± 2.3%) and strain rate were observed in paediatric patients (*n* = 40, mean age 12 ± 3 years) despite normal EF (*P* < 0.001),^79^ with similar findings extending decades post-repair in adults (*n* = 45, mean age 37 ± 9 years, *P* < 0.05).^78^ Developmental studies indicate that early pressure overload, as occurs in foetal AS or CoA, may alter myocardial fibre orientation and contribute to lifelong deformation abnormalities.^80^

##### Clinical advice

**Figure 6:**
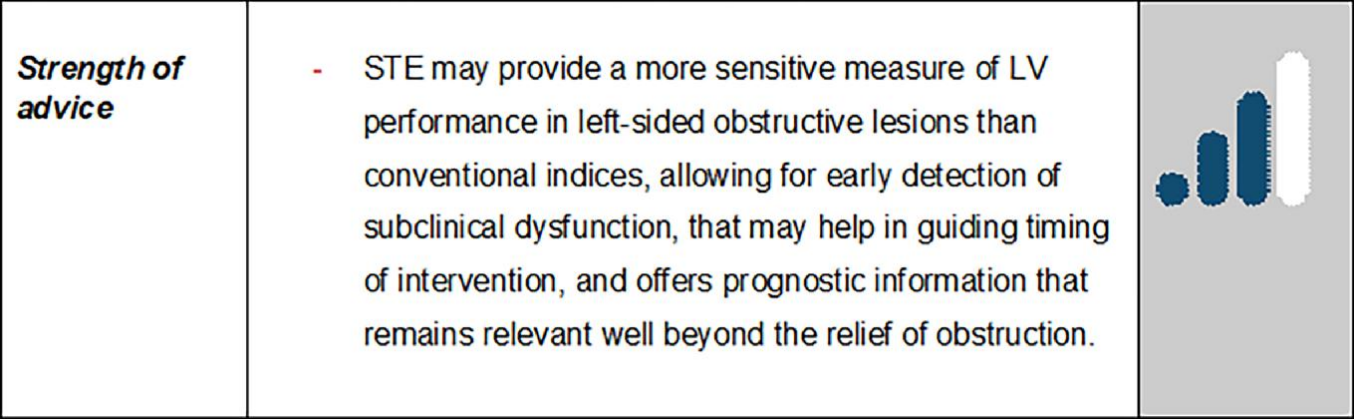
Clinical advice on application of STE in left sided obstruction (Aortic Stenosis and Coarctation of the Aorta).

**Gap in knowledge:** (*Figure 6*) long-term clinical implications of persistent strain abnormalities in left-sided obstructive lesions remain incompletely understood, particularly regarding their reversibility after relief of obstruction, their relationship with myocardial remodelling, and their potential role in guiding early intervention and long-term follow-up strategies.

#### ALCAPA

STE has proven particularly informative in patients with anomalous left coronary artery from the pulmonary artery (ALCAPA), revealing persistent and regionally heterogeneous myocardial dysfunction long after apparently ‘successful’ surgical repair and normalization of EF.^69–72^ Long-term follow-up^69^ of 18 surgically repaired ALCAPA patients (median age at repair 7 months; median echocardiographic follow-up ≈17 years) showed that although mean LVEF improved from 33 ± 17% preoperatively to 55 ± 6% at follow-up, global GLS (GLS) remained significantly reduced vs. matched controls (LV GLS −15.8 ± 3.3% vs. −21.9 ± 1.7%, *P* < 0.01), with marked regional impairment in the former LCA territory (LCA-region LS −13.8 ± 7.3% vs. RCA-region −19.0 ± 4.4%, *P* < 0.00001);^69^ right ventricular and atrial longitudinal strain were also lower and diastolic indices (E/E’) were worse in the ALCAPA cohort, demonstrating that normalization of EF does not equate to complete myocardial recovery [75].

Smaller series^70^ using a regional STE approach have shown that segmental longitudinal strain is sensitive for detecting residual coronary pathology or fibrosis after reimplantation. In a cohort of 10 asymptomatic post-reimplantation patients (median age at surgery ∼188 days; mean follow-up 8.7 ± 4.7 years) with normal EF, longitudinal and circumferential deformation were reduced in LCA perfusion territories compared with RCA territories; importantly, patients with MRI- or angiography-confirmed LCA stenosis exhibited the lowest regional longitudinal strain values (examples: −11.7%, −14.7%, −14.8%), and a mean longitudinal strain threshold >−15% in LCA segments was proposed to flag those with residual coronary disease.^70^

Larger cross-sectional studies confirm a consistent pattern.^71,72^ Di Salvo and colleagues^72^ evaluated 30 asymptomatic repaired ALCAPA patients (median age 4 years, range 1–25) with LVEF >50% and demonstrated significantly lower LV GLS (−17.6 ± 3.5% vs. −23.4 ± 3.1% in controls, *P* < 0.0001) and reduced LV torsion (9.1° ± 4.9° vs. 11.9° ± 3.3°, *P* = 0.046), while standard LV dimensions and EF were comparable to controls, again underscoring that conventional EF underestimates residual systolic and rotational abnormalities.^71,72^

Earlier deformation studies likewise reported persistent longitudinal deformation deficits after repair despite apparent recovery of radial function and EF, suggesting selective subendocardial vulnerability and chronically altered fibre mechanics in the reperfused myocardium; these early observations established the rationale for routine STE surveillance in this population.^72^

##### Clinical advice

**Figure 7:**
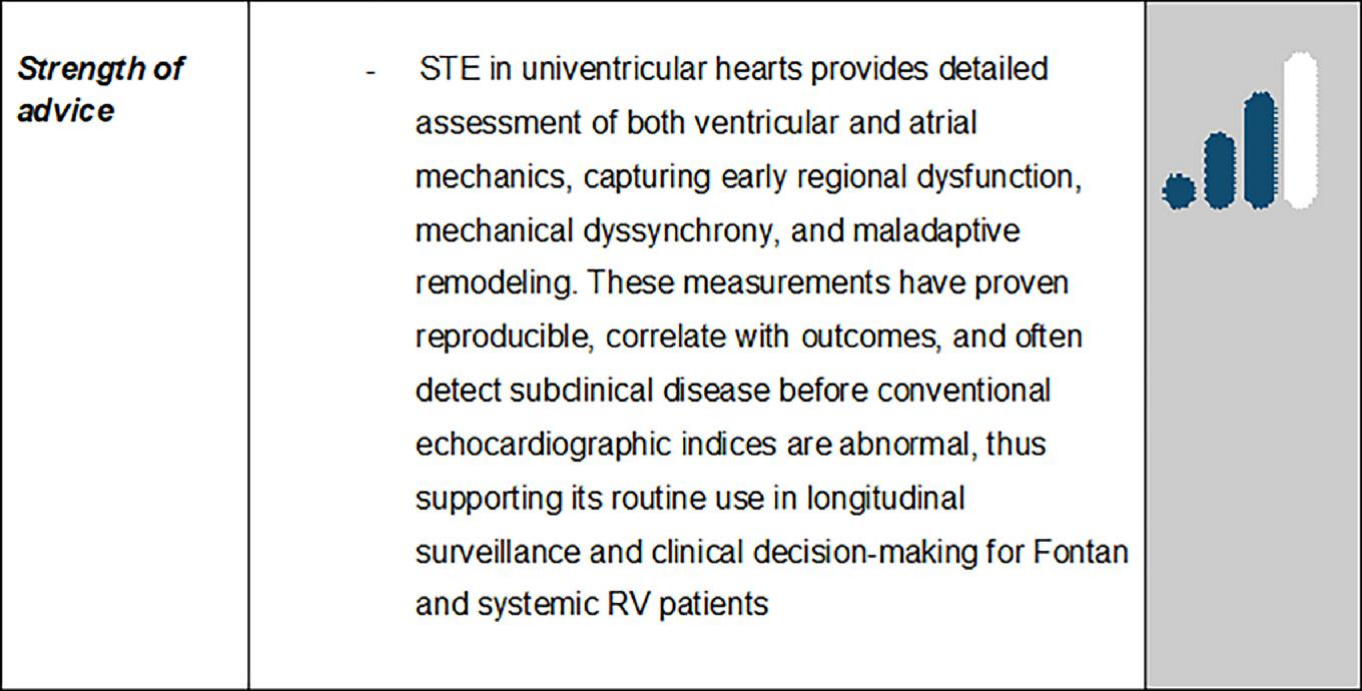
Clinical advice on application of STE in ALCAPA.

**Gap in knowledge:** (*Figure 7*) The long-term clinical significance, and prognostic implication of persistent regional strain abnormalities in repaired ALCAPA remain unclear.

#### Univentricular heart across different stages of Fontan palliation

STE has become a cornerstone for assessing myocardial mechanics in patients with single-ventricle physiology, including those with Fontan circulation or systemic RV, providing insights beyond conventional indices such as EF or FAC.^3–5^ STE allows quantification of global and regional longitudinal, circumferential, and rotational deformation, detecting early subclinical dysfunction, mechanical dyssynchrony, and ventricular–atrial interactions.^20–35^

In patients with a systemic left ventricle,^5,20,21^ longitudinal studies have consistently demonstrated progressive alterations in ventricular mechanics over time. In a cohort of 34 children with single left ventricle post-Fontan (mean age 9.2 ± 4.1 years), GLS was significantly impaired (−15.2 ± 3.0% vs. −20.1 ± 2.5% in controls, *P* < 0.001) with reduced torsion, particularly in lateral wall segments, despite preserved ejection fraction (58 ± 5%).^20^ Similar observations in 29 young Fontan patients revealed abnormal torsion patterns and regional heterogeneity in contraction, indicating impaired fibre mechanics.^21^ Longitudinal follow-up of 42 adolescents over 5 years showed progressive worsening of GLS (−16.3 ± 2.8% to −14.9 ± 3.1%, *P* < 0.05), suggesting ongoing chronic ventricular remodelling even in the presence of clinically stable haemodynamics.^5^ These data highlight the value of STE for detecting early subclinical dysfunction and monitoring long-term remodelling in systemic LVs.^5,20,21^ From a clinical perspective, these findings may influence surveillance strategies in Fontan patients. Progressive deterioration in GLS or torsional mechanics over serial evaluations may identify patients requiring closer follow-up even in the presence of stable ejection fraction and apparently compensated haemodynamics.^5,20,21,30^ STE abnormalities may be particularly relevant when accompanied by declining exercise tolerance or evidence of increased filling pressures, potentially prompting earlier comprehensive assessment including CMR, cardiopulmonary exercise testing, or invasive haemodynamic evaluation.^24,30^

In single right ventricle physiology (systemic RV),^4,22,26,27^ STE has proven highly sensitive for identifying mechanical dyssynchrony and subtle systolic impairment. In 26 paediatric patients, STE-derived strain correlated strongly with MRI-measured ejection fraction (*r* = 0.81, *P* < 0.001) and demonstrated excellent reproducibility.^4^ Activation delay–induced dyssynchrony was frequently observed in 38 systemic RV patients, with segmental strain reductions correlating with QRS prolongation (*r* = 0.53, *P* < 0.01).^27^ Application of three-dimensional STE in post-Fontan single left and right ventricles revealed impaired twist and torsion mechanics, with regional strain abnormalities predominantly affecting septal and lateral walls, which were associated with reduced exercise capacity and elevated filling pressures.^22,26^ Collectively, these findings emphasize STE’s ability to detect early mechanical derangements in systemic RVs, providing insight beyond conventional volumetric or ejection fraction assessment.^4,22,26,27^ Importantly, STE findings may complement standard echocardiography and CMR during longitudinal surveillance of single-ventricle patients, supporting individualized follow-up strategies and potentially identifying patients requiring earlier reassessment before overt ventricular dysfunction becomes apparent.^24,30^

Beyond detection, STE has provided important prognostic information in single-ventricle populations.^23,24^ In 45 hypoplastic left heart syndrome patients, reduced systemic RV longitudinal strain during the interstage period was independently associated with adverse outcomes, including hospital readmission and transplant listing (*P* < 0.01).^23^ Similarly, in 28 Fontan patients, STE demonstrated subclinical dysfunction even when EF was preserved and correlated with cardiac magnetic resonance findings, supporting its use as a diagnostic adjunct for risk stratification.^24^ These studies confirm that strain imaging can identify high-risk patients earlier than conventional metrics alone.^23,24^

Atrial mechanics are increasingly recognized as important determinants of function in single-ventricle physiology.^25,36–39^ Multiple studies have documented reductions in both left and right atrial reservoir and conduit strain in Fontan patients, which correlate with lower cardiac index, elevated filling pressures, and reduced exercise capacity.^25,36–39^ In 39 Fontan patients, left atrial strain independently predicted functional limitations, underscoring the importance of incorporating atrial STE into comprehensive surveillance strategies for single-ventricle physiology.^37^ Atrial strain assessment may have practical clinical implications because reduced reservoir and conduit function have been associated with elevated filling pressures, lower cardiac index, and impaired exercise capacity.^25,36–38^ Incorporation of atrial STE into routine longitudinal evaluation may therefore provide incremental information for identifying Fontan patients at risk of progressive circulatory failure before overt deterioration of conventional parameters occurs.^25,36–38^

##### Clinical advice

**Figure 8:**
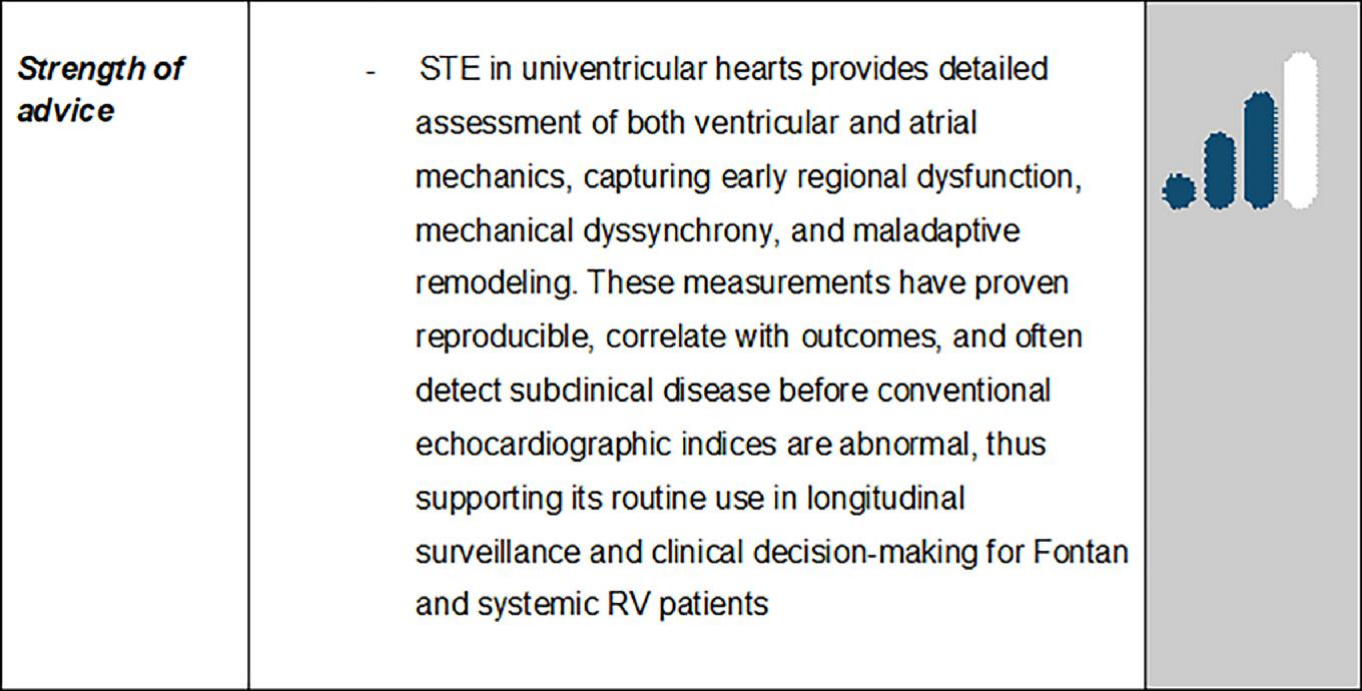
Clinical advice on application of STE in univentricular heart across different stages of Fontan palliation.

**Gap in knowledge:** (*Figure 8*) The long-term clinical and prognostic implications for persistent ventricular and atrial strain abnormalities in single-ventricle physiology remain poorly defined. Data on application of atrial STE are encouraging but thus far still limited.

#### Post paediatric cardiac surgery

##### Ventricular strain

Studies on ventricular strain after paediatric cardiac surgery are limited.^52,98–103^ In 117 children undergoing corrective cardiac surgery,^101^ STE revealed an early and marked postoperative reduction in global, basal, and mid left ventricular strain (LVε), with partial recovery over time but persistent impairment at discharge compared with preoperative values and healthy controls. In contrast, apical segments showed relative postoperative hypercontractility, while left ventricular ejection fraction (LVEF) decreased only transiently and normalized rapidly.^101^ These findings indicate that STE detects subclinical systolic dysfunction not captured by LVEF, supporting its role in postoperative monitoring.^101^ A randomized trial^102^ comparing milrinone and levosimendan in infants demonstrated similar patterns of postoperative deterioration and recovery of biventricular strain, with no significant differences between groups. Both agents provided comparable perioperative inotropic support.^102^

Preoperatively, preterm infants with PDA exhibited higher left ventricular global longitudinal strain (LVGLS) and lower right ventricular free wall strain (RVFWSL) compared with those without PDA.^103^ Following PDA ligation, LVGLS and left atrial reservoir strain (LASr) showed a transient decline with recovery within 24–48 h, whereas RVFWSL improved promptly.^103^

#### Atrial strain

Data on atrial strain response after cardiac surgery mostly derive from adult literature,^105^ where atrial strain analysis has been used in the prediction of atrial fibrillation.^104^ Data on children are extremely limited. In 31 children undergoing cardiac surgery (mean age 1.7 years) (112i), atrial strain was evaluated preoperatively and at three postoperative intervals. Across 309 examinations, all atrial strain parameters (contractile, conduit, reservoir) were significantly reduced compared with 131 healthy controls (*P* < 0.0001).^105^ The lowest values occurred within 24–36 h after surgery, with only partial recovery thereafter; at discharge, atrial strain remained markedly depressed (*P* < 0.0001). Atrial strain correlated with bypass duration, ventricular strain, and ejection fraction (*P* < 0.05), indicating coupled atrial–ventricular dysfunction in the early postoperative period.^105^

### Heart transplant

Despite major advances in surgical techniques and immunosuppression, post-transplant complications such as graft rejection, cardiac allograft vasculopathy (CAV), and progressive ventricular dysfunction remain key determinants of morbidity and mortality in paediatric heart transplant recipients.^25,105–113^ Conventional echocardiographic indices, including ejection fraction and fractional shortening, often fail to detect early or subclinical graft dysfunction, whereas STE provides a sensitive, quantitative assessment of myocardial deformation (strain) and mechanics, allowing earlier recognition of subtle alterations in graft performance.^25,105–113^ Using two-dimensional STE, studies have shown that paediatric heart transplant recipients (*n* = 45, mean age 13.1 ± 3.4 years) exhibit significantly reduced global longitudinal strain (GLS: −17.2 ± 2.5%) compared with healthy controls (−21.6 ± 2.0%, *P* < 0.001), despite preserved LVEF. GLS correlates inversely with time since transplantation (*r* = −0.52, *P* = 0.003), suggesting progressive subclinical myocardial dysfunction over time.^108^ These findings underscore GLS as a sensitive and reproducible marker of graft health beyond conventional systolic indices.^25,105–108^ From a clinical perspective, serial reductions in GLS during follow-up may identify transplant recipients requiring intensified surveillance even when conventional systolic indices remain preserved.^106,108,109^ STE-derived abnormalities may be particularly useful in patients with discordance between symptoms and standard echocardiographic findings, potentially prompting earlier comprehensive evaluation including endomyocardial biopsy, CMR, or closer clinical monitoring.^106,108^

In parallel, circumferential strain has been associated with acute rejection episodes. In a cohort of 28 paediatric heart transplant patients (median age 14 years), those with biopsy-proven acute cellular rejection (*n* = 8) demonstrated significantly lower circumferential strain (−12.9 ± 2.4%) compared with stable transplant recipients (−18.1 ± 2.2%, *P* < 0.001). A circumferential strain cut-off of −15.0% yielded an area under the receiver operating characteristic curve (AUC) of 0.87 for rejection detection, suggesting that STE may serve as a non-invasive adjunct to endomyocardial biopsy in the surveillance of rejection.^106^ Importantly, STE is unlikely to replace endomyocardial biopsy but may function as a complementary screening tool capable of identifying patients requiring more intensive diagnostic assessment.^105,106^ Integration of strain findings with clinical status, biomarkers, and biopsy results may improve sensitivity for detecting early graft dysfunction or rejection before overt deterioration develops. Similarly, in 41 paediatric transplant recipients (mean age 15.2 ± 3.8 years), strain-derived pressure–strain loop analysis was used to assess myocardial work efficiency. Patients with angiographically confirmed CAV (*n* = 12) had significantly lower global myocardial work efficiency (GWE: 84 ± 6%) compared to those without CAV (91 ± 4%, *P* < 0.001), with a strong correlation between GWE and vasculopathy severity (*r* = −0.62, *P* < 0.001), supporting the role of STE-derived myocardial work indices as novel, load-adjusted, and non-invasive markers for CAV detection and grading.^107^ These findings suggest potential clinical utility for identifying transplant recipients who may benefit from earlier vasculopathy screening or closer longitudinal surveillance. Integration with coronary imaging and CMR may further improve characterization of myocardial injury and disease progression.^107^

Further insights into graft function and adaptation have been obtained through biventricular and longitudinal analyses. Three-dimensional STE evaluation of 36 clinically stable paediatric transplant recipients (mean age 11.6 ± 3.2 years) revealed significantly reduced left ventricular GLS (−18.5 ± 2.6%) and right ventricular free wall strain (−21.7 ± 3.4%) compared with age-matched controls (*P* < 0.01), despite preserved ejection fraction, indicating subclinical biventricular dysfunction even in asymptomatic patients.^110^ Serial follow-up studies of 32 recipients (mean age 12.7 ± 2.9 years) demonstrated a progressive improvement in GLS from −15.8 ± 2.1% at 1 month to −18.9 ± 2.5% at 12 months post-transplant (*P* = 0.002), consistent with normalization of ventricular geometry and donor heart adaptation to the paediatric thoracic cavity.^112^ Similarly, in 27 rejection-free children (mean age 14.3 ± 3.7 years), right ventricular free wall strain improved significantly from −19.4 ± 3.2% to −23.1 ± 2.9% during the first year (*P* = 0.01), correlating with improved exercise tolerance (*r* = 0.44, *P* = 0.02), highlighting the ability of STE to track dynamic graft remodelling and functional recovery over time.^105^

Diastolic mechanics also appear to be affected in this population. In a pilot study including 15 paediatric transplant recipients (mean age 12.1 ± 3.6 years), STE-derived strain rate imaging revealed increased ventricular stiffness and reduced early diastolic strain rate (1.2 ± 0.3 vs. 1.9 ± 0.4 s^−1^, *P* = 0.002) compared with controls, despite normal Doppler-derived filling pressures, indicating that STE can detect subclinical diastolic dysfunction before conventional indices become abnormal.^110^ These data reinforce the role of strain-based imaging in identifying early myocardial mechanical alterations that may precede overt graft dysfunction.^109–111^ Although STE abnormalities frequently precede changes in EF, validated paediatric thresholds for therapeutic intervention remain incompletely established.^25,106–112^ Consequently, isolated strain abnormalities should currently be interpreted primarily as markers for intensified surveillance and serial reassessment rather than standalone triggers for treatment escalation. Persistent deterioration over time or concomitant abnormalities across multiple parameters may justify earlier multimodality investigation.

Additional insights from related paediatric populations further support the diagnostic and monitoring value of STE. In 18 paediatric patients supported with left ventricular assist devices, progressive reductions in strain indices were observed with increasing support duration, reflecting the relationship between strain and altered ventricular loading conditions.^112^ Likewise, in children undergoing bilateral lung transplantation for pulmonary arterial hypertension, right ventricular strain normalized after surgery, demonstrating the ability of STE to capture recovery of myocardial mechanics in the setting of advanced cardiopulmonary disease.^113^

Taken together, these findings across multiple paediatric cohorts^25,105–113^ establish STE as a comprehensive, non-invasive modality capable of detecting early systolic and diastolic dysfunction, monitoring rejection and vasculopathy, and tracking graft remodelling and adaptation over time. Incorporating STE-derived parameters into post-transplant surveillance protocols could significantly enhance early diagnosis, guide therapeutic decisions, and improve long-term outcomes in paediatric heart transplant recipients.^25,105–113^ Importantly, STE should be considered complementary to conventional echocardiography, endomyocardial biopsy, and CMR, supporting individualized surveillance strategies and potentially identifying patients requiring closer monitoring before overt graft dysfunction develops.^25,106–112^

#### Clinical advice

**Figure 9:**
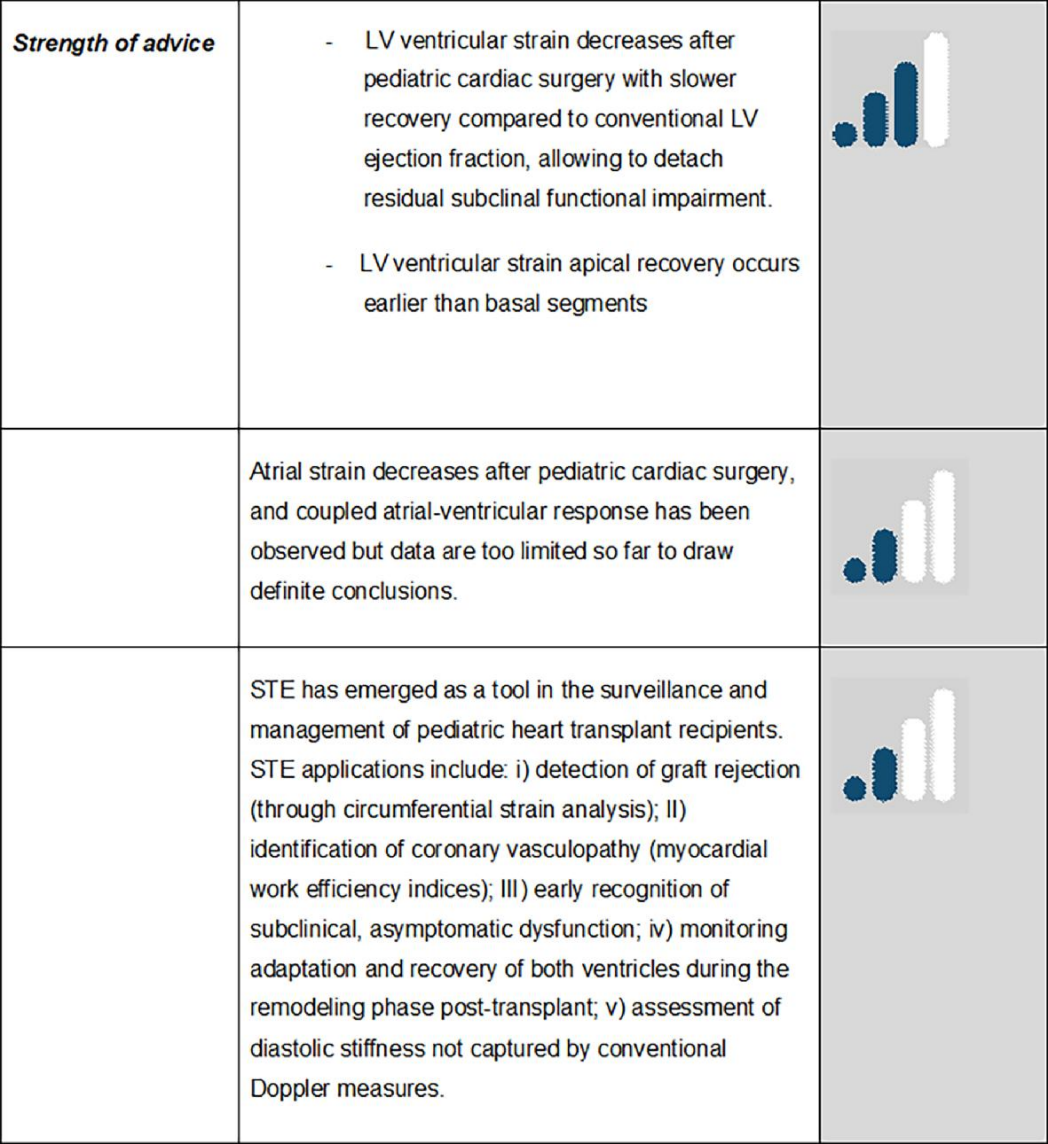
Clinical advice on application of STE in post paediatric cardiac surgery.

**Gap in knowledge:** (*Figure 9*) The long-term clinical and prognostic values of STE applications after paediatric and congenital cardiac surgery remain limited.

#### Paediatric rhythm disorders including heart block, pacing, and dyssynchrony

Rhythm disturbances in children, including congenital heart block, Wolff–Parkinson–White (WPW) syndrome, and pacing-induced ventricular dyssynchrony, represent a major clinical challenge in paediatric cardiology, with potential long-term consequences including ventricular remodelling, systolic dysfunction, and heart failure.^115–128^ Conventional echocardiographic indices often fail to detect early or subtle alterations in myocardial mechanics, whereas STE provides a sensitive and quantitative assessment of myocardial deformation and mechanical synchrony, allowing earlier identification of dysfunction and guiding therapeutic interventions.^115–128^

In WPW syndrome, STE has been shown to detect both global and regional mechanical abnormalities associated with accessory pathways. In a cohort of 45 children (mean age 12.4 ± 3.2 years) compared with 30 healthy controls, GLS was significantly reduced (−18.6 ± 2.3% vs. −21.9 ± 2.1%, *P* < 0.001) and mechanical dyssynchrony increased (standard deviation of time-to-peak strain: 42 ± 12 ms vs. 28 ± 8 ms, *P* = 0.002), with regional strain abnormalities corresponding to the location of the accessory pathway.^115^ Improvements in GLS and synchrony were observed after ablation, highlighting the utility of STE not only for assessing the mechanical consequences of preexcitation but also as an adjunct tool for localization of accessory pathways.^115^

In children with congenital complete heart block requiring chronic pacing, STE has provided important insights into the impact of pacing sites on ventricular mechanics. In 28 patients (mean age 9.7 ± 4.1 years), those receiving LV epicardial pacing (*n* = 15) demonstrated superior myocardial mechanics compared with RV)septal pacing (*n* = 13), with GLS of −20.4 ± 2.7% vs. −16.9 ± 3.1% (*P* < 0.01) and lower mechanical dispersion (36 ± 10 ms vs. 54 ± 14 ms, *P* < 0.01), while higher pacing burden correlated with worsening GLS (*r* = −0.63, *P* < 0.001).^117^ Long-term follow-up of 36 children (mean implantation age 3.1 ± 1.4 years, mean follow-up 8.2 years) demonstrated progressive dyssynchrony (inter-segmental delay from 32 ± 9 ms to 57 ± 15 ms, *P* < 0.01), preceding overt decline in ejection fraction, confirming that prolonged non-physiological pacing induces early subclinical dysfunction detectable by STE.^118^

The importance of pacing sites in preserving synchrony has been further demonstrated in infants and older children. In 25 infants (mean age 9.2 ± 3.5 months) undergoing LV epicardial pacing, follow-up over 5 years revealed preserved synchrony and favourable strain parameters (GLS −21.1 ± 2.5%, mechanical dispersion 34 ± 12 ms), significantly superior to historical RV pacing cohorts (*P* < 0.05).^119^ Similarly, in 30 children (mean age 11.5 ± 3.9 years), left bundle branch area pacing (LBBAP, *n* = 15) provided lower inter-segmental delay (27 ± 6 ms vs. 49 ± 11 ms, *P* < 0.001) and higher GLS (−21.9 ± 2.0% vs. −18.4 ± 2.8%, *P* = 0.004) than RV septal pacing (*n* = 15), with synchrony indices correlating positively with ejection fraction (*r* = 0.58, *P* = 0.01).^120^ These findings indicate that physiological pacing techniques, guided by STE-derived mechanical metrics, can reduce long-term dyssynchrony and preserve ventricular function in paediatric populations.^117–120^

Abnormal mechanical patterns associated with pacing have also been characterized using normative data. Reference values^125^ for LV rotation and torsion in 174 healthy children (0–20 years), which serve as essential benchmarks for detecting pathological alterations, have been established. Acute AV-synchronous RV pacing in 18 children (mean age 13.6 ± 2.8 years) induced immediate reductions in GLS (from −22.3 ± 2.4% to −19.1 ± 2.7%, *P* < 0.01) and increased mechanical dispersion (from 31 ± 9 ms to 47 ± 11 ms, *P* < 0.01), demonstrating that even short-term non-physiological pacing can provoke dyssynchrony.^124^ Moreover, STE has been instrumental in optimizing cardiac resynchronization therapy (CRT) post-congenital heart surgery, with regional strain analysis guiding lead placement and programming, resulting in improved synchrony and systolic performance.^126,128^ These observations reinforce STE’s role in both the detection and correction of pacing-induced mechanical remodelling.^124–128^

STE has further demonstrated value in rhythm-related myocardial dysfunction beyond pacing. In 22 children (mean age 7.9 ± 3.1 years) with tachycardia-induced cardiomyopathy secondary to chronic atrial tachycardia, GLS was markedly impaired (−15.6 ± 3.4%) with elevated mechanical dispersion (62 ± 13 ms) at baseline. Following rhythm control, GLS and dispersion improved significantly (−20.8 ± 2.7%, *P* < 0.001; 37 ± 9 ms, *P* < 0.001), correlating with recovery of left ventricular function (*r* = 0.67, *P* < 0.01),^122^ illustrating STE’s role in both detection and monitoring of functional recovery.^120–122^

Finally, STE has applicability in complex congenital heart disease and single-ventricle physiology. In a 14-year-old Fontan patient with failing circulation, STE identified marked interventricular and intraventricular dyssynchrony pre-CRT, which normalized post-implantation alongside improved systemic ventricular performance.^123^ Although limited to case-level evidence, this underscores the utility of STE in guiding resynchronization therapy in challenging anatomical settings.^123^

##### Clinical advice

**Figure 10:**
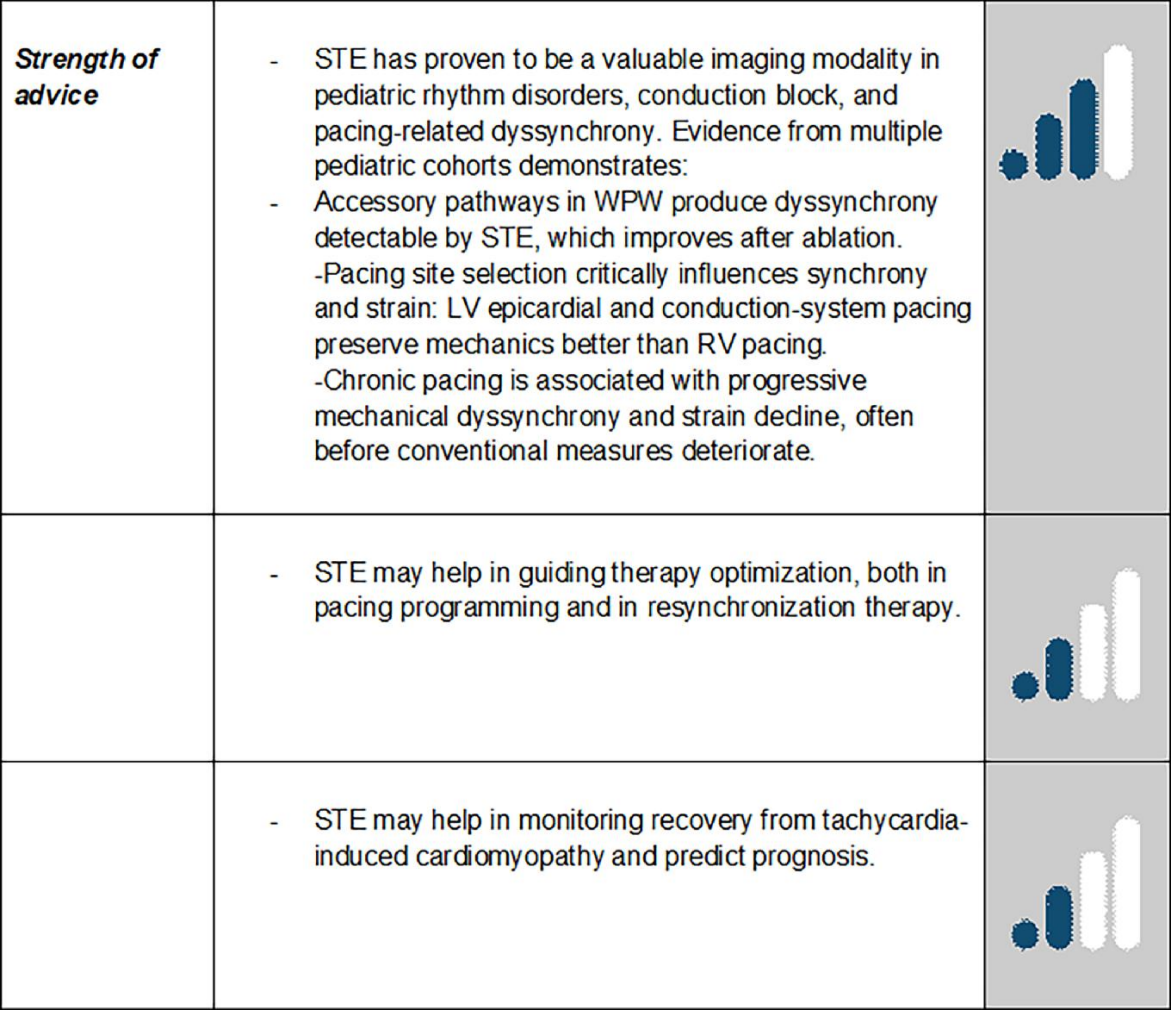
Clinical advice on application of STE in paediatric rhythm disorders, including heart block, pacing, and dyssynchrony.

**Gaps in knowledge:** (*Figure 10*) Despite multiple evidence proving its utility, the use of STE in paediatric rhythm disorders needs to be standardized and incorporated into management flow charts. When and how to monitor need to be standardized, and defined thresholds for intervention to prevent progressive ventricular dysfunction need to be established.

#### Paediatric cardiomyopathy

STE has emerged as a highly sensitive tool for detecting subclinical myocardial dysfunction in paediatric cardiomyopathies, often revealing early impairments that are not apparent on conventional indices such as LVEF or FAC.^129–135^ STE provides quantitative assessment of global and regional myocardial deformation, including longitudinal, circumferential, radial strain, and rotational mechanics, allowing for detailed mapping of regional contractile deficits.^129–135^

In hypertrophic cardiomyopathy (HCM), studies consistently report reductions in GLS even when EF remains preserved.^129^ In a cohort of 42 children (mean age 11.2 ± 3.8 years),^129^ GLS was −15.4 ± 3.1% vs. −20.3 ± 2.4% in controls (*P* < 0.001), while EF remained within the normal range (60 ± 5%, *P* = NS). Regional analysis revealed pronounced basal septal strain reduction, reflecting the hypertrophic pattern.^135^ Similarly, in 37 paediatric HCM patients (mean age 12.5 ± 4.2 years), increased LA stiffness, assessed by atrial reservoir strain (21.3 ± 5.4%, *P* < 0.001 vs. controls), correlated with adverse cardiac events (*R* = 0.62, *P* < 0.01), highlighting the incremental prognostic value of atrial STE over conventional EF and diastolic indices.^129^

In ACM (formerly arrhythmogenic right ventricular cardiomyopathy, ARVC) form, 28 adolescents (mean age 14 ± 2.5 years)^132^ showed reduced right ventricular GLS (−17.2 ± 2.8%) with regional mechanical dispersion of the free wall segments (*P* < 0.01 vs. controls), despite preserved FAC (44 ± 6%, *P* = NS), underscoring the superiority of STE for detecting early regional abnormalities and discriminating physiological remodelling in healthy athletes from pathological changes.^132^

For metabolic and systemic cardiomyopathies, including type 1 diabetic cardiomyopathy, 50 children (mean age 12.3 ± 2.9 years)^131^ demonstrated impaired LV longitudinal strain (−16.8 ± 2.7%, *P* < 0.001 vs. controls) while EF remained preserved (61 ± 4%, *P* = NS). Regional analysis showed reduced strain predominantly in the mid-lateral and basal inferoseptal segments, indicating early regional dysfunction.^131^

In septic cardiomyopathy, 34 critically ill children (mean age 7.9 ± 3.6 years) had GLS −14.9 ± 3.2% (*P* < 0.001 vs. healthy controls) with preserved EF (58 ± 6%, *P* = NS), and regional strain analysis revealed more pronounced dysfunction in the interventricular septum, highlighting heterogeneity of myocardial depression in systemic inflammation.^131^ Blood speckle-tracking in paediatric patients also detected early diastolic intraventricular pressure differences, showing abnormal relaxation patterns even in the absence of EF reduction (*P* < 0.01).^134^

The integration of pressure–strain loops has provided a novel, load-independent assessment of myocardial performance.^6,133^ In 30 children (mean age 11 ± 4 years) with various cardiomyopathies, myocardial work efficiency was reduced (*P* < 0.01 vs. controls) despite preserved EF, with regional deficits in the septal and lateral walls correlating with impaired longitudinal strain (*R* = 0.71, *P* < 0.001).^133^

Overall, STE allows detection of both global and regional myocardial impairment, often preceding EF or FAC reduction, and provides quantitative data for early diagnosis, risk stratification, and longitudinal follow-up in paediatric cardiomyopathies of diverse aetiologies.^129–135^

In paediatric HCM, LA^135^ mechanics have emerged as sensitive indicators of diastolic dysfunction. In a cohort of 78 children and young adults (ages 3–25 years) with either phenotype-positive HCM (P+, *n* = 46) or genotype-positive, phenotype-negative HCM (G + P−, *n* = 32), compared with 20 healthy controls, STE revealed that P+ patients exhibited significantly impaired LA conduit (36% vs. 48%, *P* < 0.001) and reservoir function (137% vs. 180%, *P* < 0.001), whereas contractile function was preserved (31% vs. 32%, *P* = 0.87). Phasic LA strain was also markedly reduced in P+ patients, including four-chamber LASr (29% vs. 41%, *P* < 0.001), two-chamber LASr (30% vs. 41%, *P* < 0.001), average LASr (29% vs. 42%, *P* < 0.001), and LA contractile strain (9% vs. 12%, *P* = 0.016). In the subset undergoing exercise testing (*n* = 35), LA conduit function correlated modestly but significantly with peak aerobic capacity (VO_2_ max, *r* = 0.42, *P* = 0.019), indicating that LA dysfunction contributes to functional limitation in paediatric HCM.^135^

Recent evidence also highlights the role of atrial deformation imaging to assess diastolic function in paediatric cardiomyopathies. In a cohort of 136 children with DCM, HCM, and RCM, LA strain and strain rate showed a progressive decline with worsening diastolic dysfunction and demonstrated excellent diagnostic accuracy for distinguishing cardiomyopathy from healthy controls (AUC 0.976 and 0.946). Unlike conventional Doppler indices, LA strain parameters effectively differentiated degrees of diastolic impairment, supporting their use as sensitive markers of diastolic dysfunction in paediatric CM populations.^212^

##### Clinical advice

**Figure 11:**
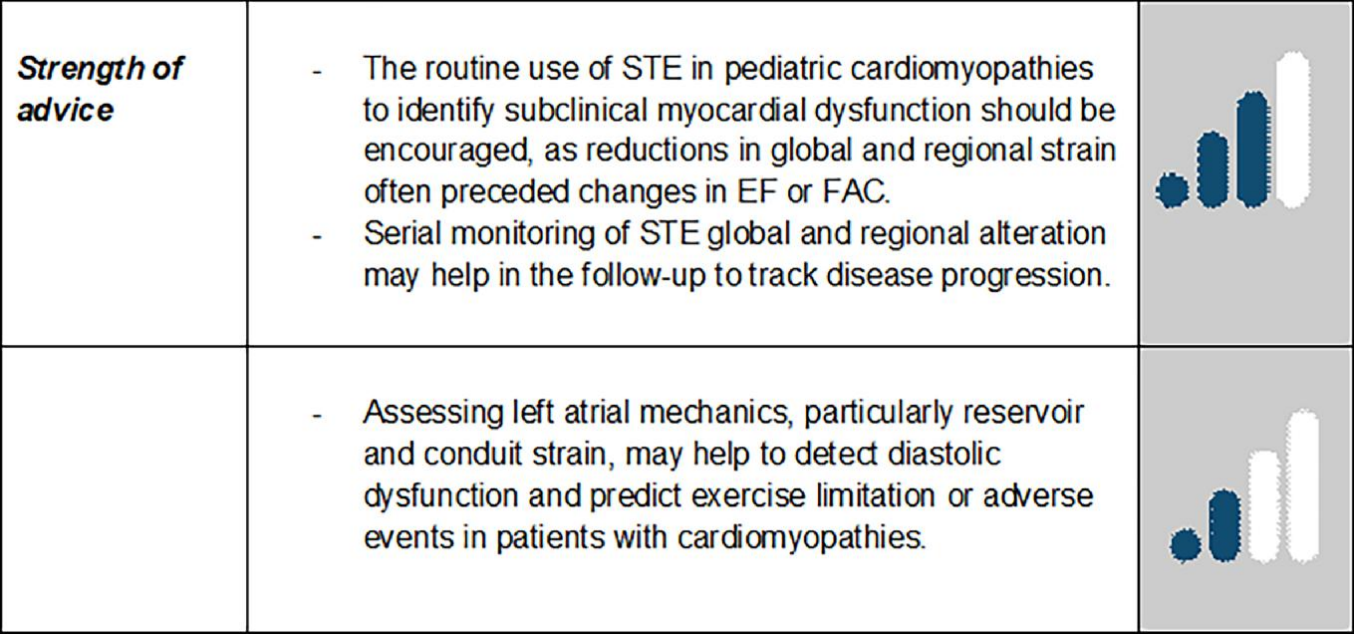
Clinical advice on application of STE in paediatric cardiomyopathy.

**Gaps in knowledge:** (*Figure 11*) The long-term prognostic significance and optimal thresholds of STE-derived strain parameters in paediatric cardiomyopathies remain poorly defined, limiting standardized guidance for clinical decision-making.

#### Paediatric cardiotoxicity

Cardiotoxicity is one of the most significant late effects of paediatric cancer therapy, particularly among survivors exposed to anthracyclines, high-dose alkylating agents, or chest-directed radiotherapy.^135–148^ Conventional echocardiographic indices such as LVEF are relatively insensitive for detecting early myocardial dysfunction.^135–148^ STE, and particularly GLS, has emerged as a sensitive imaging biomarker capable of identifying subclinical myocardial impairment prior to the onset of overt cardiomyopathy.^136–149^ Prospective paediatric oncology studies consistently show that STE identifies subclinical myocardial dysfunction earlier than conventional parameters, with cohorts of 102 children with haematologic malignancies demonstrating significant GLS impairment correlated with higher anthracycline exposure and biomarker elevation (*P* < 0.001),^135^ and companion analyses of 118 patients showing that cancer type and treatment protocol influenced myocardial impairment, with GLS more sensitive than EF (*P* < 0.05).^135^ In 45 children with acute lymphoblastic leukaemia, GLS impairment occurred after the second anthracycline cycle, even when EF remained preserved (*P* < 0.01),^141^ while in 92 children persistent strain abnormalities were detected at anthracycline doses considered within ‘safe’ limits (*P* < 0.001).^138^ Long-term survivorship studies have confirmed these findings, with 52 survivors showing that nearly one-third had abnormal GLS despite normal EF (*P* < 0.01),^142^ and systematic reviews of >1200 survivors consistently demonstrating that STE is superior to conventional echocardiography for detecting asymptomatic systolic dysfunction.^143^ Large multicentre cohorts (>600 survivors) reported reduced GLS in anthracycline-exposed children, correlating with cumulative dose and younger age at exposure (*P* < 0.001),^144^ and additional analyses identified subtle GLS changes associated with vincristine exposure, suggesting polychemotherapy cardiotoxicity.^145^ GLS was the most frequent abnormality detected during screening in 102 Finnish survivors (*P* < 0.01).^147^ These findings have important implications for clinical surveillance strategies. Detection of early GLS deterioration during treatment or follow-up may identify children requiring closer cardiologic monitoring even when conventional indices such as EF remain preserved.^135,138,146,147^ In particular, serial strain assessment may be useful in patients with higher cumulative anthracycline exposure, younger age at treatment, biomarker elevation, or combined chemotherapy regimens, helping refine risk-adapted follow-up approaches.^135,143–147^ The prognostic value of STE has been validated in large survivorship cohorts, with >1500 paediatric cancer survivors showing that GLS impairment combined with NT-proBNP significantly improved cardiomyopathy risk prediction (*P* < 0.001),^146^ and meta-analytic evidence from 18 studies including ≈1100 patients confirmed consistent GLS reduction across populations, with a pooled mean difference of −2.5% between exposed and control groups (*P* < 0.001).^140^ Guidelines for late effects surveillance emphasize echocardiographic monitoring for cardiomyopathy but highlight the need for normative paediatric strain data,^147^ and while GLS is not yet universally included in routine surveillance, the accumulated evidence supports its integration into clinical practice. Despite these advances, the reversibility and long-term significance of early GLS abnormalities remain incompletely understood, and thresholds for intervention based on strain are not yet fully defined. Importantly, STE should not be interpreted in isolation but integrated with biomarkers and multimodality imaging approaches. Combined assessment using GLS and NT-proBNP significantly improved prediction of late cardiomyopathy risk compared with conventional measures alone.^145^ Similarly, STE may complement CMR, particularly in patients with equivocal findings or suspected of myocardial injury despite preserved ventricular function.^136,145^ Although strain abnormalities consistently precede reductions in EF, universally accepted paediatric thresholds for intervention are still lacking.^138,146^ Consequently, isolated GLS impairment should currently be interpreted as a signal for intensified surveillance rather than as a standalone trigger for treatment modification.^138,146^ Serial worsening over time, particularly when associated with biomarker elevation or cumulative treatment burden, may warrant earlier comprehensive reassessment.^145,146^ Overall, STE has become a cornerstone of paediatric cardio-oncology, allowing early detection of myocardial impairment, correlating with cumulative anthracycline dose and biomarkers, and providing prognostic information for late cardiomyopathy.^135–148^ Importantly, STE-derived strain assessment may complement conventional echocardiography, biomarkers, and CMR during longitudinal surveillance, supporting individualized risk stratification and potentially identifying patients requiring closer follow-up before overt ventricular dysfunction develops.^136,145–147^

##### Clinical advice

**Figure 12:**
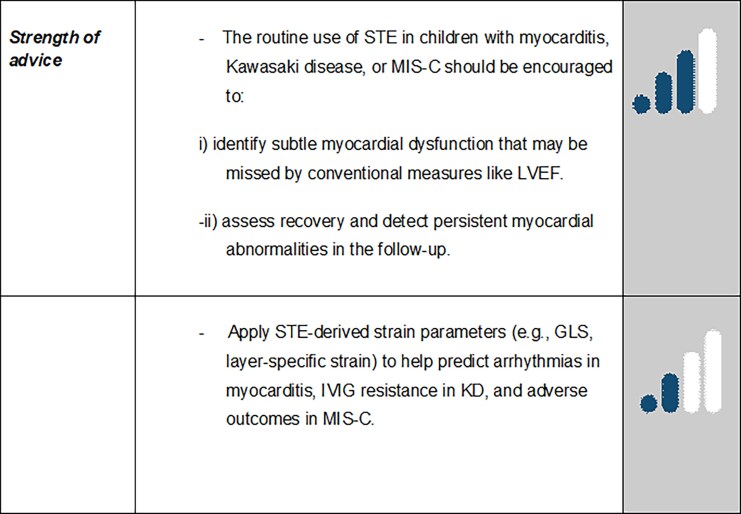
Clinical advice on application of STE in paediatric cardiotoxicity.

**Gaps in knowledge:** (*Figure 12*) the long-term significance, reversibility, or intervention thresholds for early GLS abnormalities in paediatric cancer survivors, are not well defined yet.

#### Inflammatory disease (myocarditis, Kawasaki, MISC)

Inflammatory cardiac diseases in children, particularly acute myocarditis, Kawasaki disease (KD), and multisystem inflammatory syndrome in children (MIS-C), remain critical causes of morbidity, hospitalization, and long-term cardiovascular complications.^150–163^ Conventional echocardiographic measures such as LVEF often fail to detect subtle myocardial dysfunction, particularly in early or localized diseases,^150–163^ whereas STE offers a more sensitive means of identifying early myocardial involvement and may hold prognostic significance.^150–163^ In paediatric myocarditis, the multicentre MYKKE study,^154^ which enrolled 175 patients (median age 15 years), showed that layer-specific left ventricular strain correlated strongly with LVEF (*r* > 0.8, *P* < 0.001), and abnormal longitudinal strain predicted the occurrence of arrhythmias even in patients with preserved EF (*P* < 0.001), with cut-offs of −18.0% for endocardial and −17.0% for myocardial strain providing optimal diagnostic performance (sensitivity 0.69, specificity 0.94). Smaller single-centre studies reported that children developing arrhythmias had significantly reduced GLS (−8.9% vs. −13.7%, *P* = 0.038), with basal inferior and septal regions particularly impaired (*P* = 0.009),^150,157,159^ and that GLS was markedly reduced during the acute phase of viral myocarditis (−14.5 ± 2.5% vs. −20.1 ± 1.9% in controls, *P* < 0.001) with normalization only in those with full clinical recovery (*P* < 0.05).^150^ STE abnormalities also corresponded to myocardial oedema and fibrosis on CMR imaging in children with focal myocarditis and preserved LVEF (*P* < 0.05),^157^ highlighting the ability of STE to detect localized injury not apparent on conventional measures. In KD, early paediatric studies reported significant impairment of GLS (17.0% vs. 20.2%, *P* < 0.001) and strain rate (1.66 vs. 1.97 s^−1^, *P* < 0.001) even in patients without coronary artery aneurysms,^160,161^ and larger cohorts confirmed reduced global and layer-specific strain values in the acute phase (*P* < 0.001).^160,161^ GLS improved significantly after intravenous immunoglobulin (IVIG) therapy (−15.9% to −19.6%, *P* < 0.001) in some cohorts,^152^ although persistent reduction was noted in approximately one-third of patients 12–18 months after the acute illness (*P* < 0.05).^153^ STE also identifies IVIG-resistant KD, with lower GLS values (−16.7% vs. −19.8%, *P* < 0.01) correlating with NT-proBNP levels (*r* = −0.61, *P* < 0.001).^158^ In MIS-C, studies consistently demonstrate that strain abnormalities are more prevalent and sensitive than reductions in LVEF. In a cohort of 34 children, left ventricular longitudinal (*P* < 0.001), circumferential (*P* < 0.001), and left atrial strain (*P* = 0.014) were markedly reduced, with longitudinal strain correlating with troponin (*P* = 0.005) and EF (*P* = 0.002).^162^ In the large multicentre MUSIC study, which included 349 patients, strain abnormalities were observed in 45% of patients vs. 35% with reduced EF (*P* < 0.001).^162^ The nadir of strain occurred around day five of illness, with normalization in 95% of cases within 50 days, and admission strain predicted adverse outcomes, with four-chamber LVLS associated with increased odds of cardiovascular events [*P* = 0.002, adjusted OR 1.09 per unit (95% CI 1.07–1.12)] and mid-circumferential strain showing similar predictive value (*P* = 0.001).^162^ Smaller studies confirm these findings, showing abnormal GLS in 72% of MIS-C patients while only 39% had reduced EF (*P* < 0.001),^156^ and 24% of patients continued to demonstrate abnormal GLS 6 to 12 months after the acute illness, correlating with peak troponin T (*r* = −0.52, *P* = 0.002).^153^ Case reports illustrate that STE may reveal silent myocardial impairment not detected by conventional methods.^155^ Despite these consistent findings, most studies remain small, single-centre cohorts, with the notable exceptions of the MYKKE registry^150^ and MUSIC study,^164^ limiting statistical power and external validation of prognostic thresholds such as GLS cut-offs. The dynamic course of inflammatory diseases complicates interpretation, as strain values may fluctuate rapidly, and few studies incorporate standardized longitudinal protocols. While some studies have compared STE directly to CMR-defined oedema and fibrosis, validation in larger paediatric cohorts remains sparse, and the long-term clinical relevance of persistent STE abnormalities is not yet clear. Currently, no clinical guidelines incorporate STE into therapeutic algorithms, whether for identifying IVIG-resistant KD, stratifying arrhythmic risk in myocarditis, or determining long-term surveillance needs after MIS-C.^150–163^

##### Clinical advice

**Figure 13:**
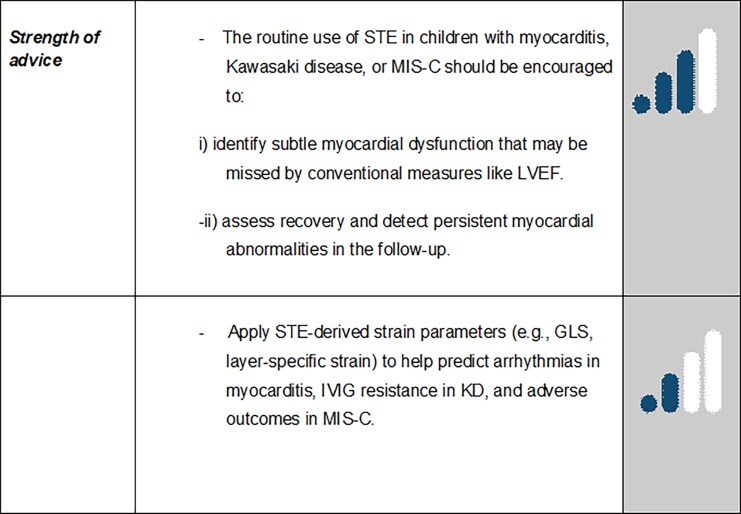
Clinical advice on application of STE in inflammatory disease (myocarditis, Kawasaki, MISC).

**Gaps in knowledge:** (*Figure 13*) The optimal thresholds, timing, and long-term clinical and prognostic significance of STE abnormalities in paediatric inflammatory cardiac diseases remain unclear. Standardized longitudinal protocols and large multicentre validations are still lacking.

#### Pulmonary hypertension

Pulmonary hypertension (PH) in children is a progressive disease characterized by increased pulmonary vascular resistance, RV pressure overload, and ultimately RV failure.^81–94^ Early recognition of subtle myocardial dysfunction is crucial to optimize management and prognosis.^81–94^ Conventional echocardiographic parameters, such as TAPSE and FAC, have limitations in sensitivity and reproducibility in the paediatric population,^81–94^ whereas STE has emerged as a sensitive and angle-independent imaging modality that quantifies myocardial deformation and provides novel insights into ventricular–atrial coupling in paediatric PH.^81–94^ Several studies have demonstrated that RV longitudinal strain is significantly impaired in children with PH compared with healthy controls.^82–94^ In prospective paediatric cohorts, RV free wall longitudinal strain was markedly reduced (−16.8 ± 3.9% vs. −23.4 ± 2.7%, *P* < 0.001), correlating with mean pulmonary artery pressure (*r* = −0.71, *P* < 0.001) and pulmonary vascular resistance index (r = −0.68, *P* < 0.001).^82^ RV strain has also been shown to predict clinical deterioration, with cut-offs around −18% associated with adverse outcomes (sensitivity 85%, specificity 81%).^94^ Correlations between RV strain, TAPSE (*r* = 0.72, *P* < 0.001), and 6-min walk distance (*r* = 0.65, *P* = 0.002) have been reported, supporting STE as a reliable marker of RV function even in complex congenital heart disease-associated PH.^86^ The RA plays a critical role in modulating RV preload in PH,^95–97^ and STE enables quantification of RA reservoir, conduit, and contractile strain. Reduced RA reservoir strain (20.3 ± 5.6% vs. 34.1 ± 6.7%, *P* < 0.001) independently predicted transplant-free survival,^97^ and RA reservoir strain correlated inversely with right atrial pressure (*r* = −0.69, *P* < 0.001), outperforming conventional indices in predicting clinical worsening.^95–97^ Beyond isolated RV analysis, STE allows assessment of biventricular interaction, with studies reporting impaired both RV and LV longitudinal strain in chronic PH, highlighting ventricular interdependence.^88,91^ Diminished RV strain at diagnosis in infants with bronchopulmonary dysplasia-related PH was associated with increased mortality risk (hazard ratio 2.5, *P* = 0.01).^91^ STE has also been applied in neonates with congenital diaphragmatic hernia-related PH,^84,203–205^ showing improved RV strain after treatment (from −14.1 ± 3.2% to −18.3 ± 2.5%, *P* = 0.004), paralleling haemodynamic improvement,^84^ and detecting early biventricular dysfunction as a biomarker of disease severity and ventricular interdependence.^204–206^ Advanced applications include blood speckle tracking to visualize pulmonary artery flow, with abnormal vortex formation correlating with disease severity (*r* = 0.61, *P* = 0.003),^75^ and validation of STE-derived indices with cardiac MRI.^87^ Emerging technologies such as three-dimensional STE^92^ and machine-learning–enhanced strain analysis may further refine non-invasive assessment of paediatric PH, and integration of atrial and ventricular strain metrics into paediatric PH risk scores could improve prognostic stratification and treatment monitoring.^81–94^

##### Clinical advice

**Figure 14:**
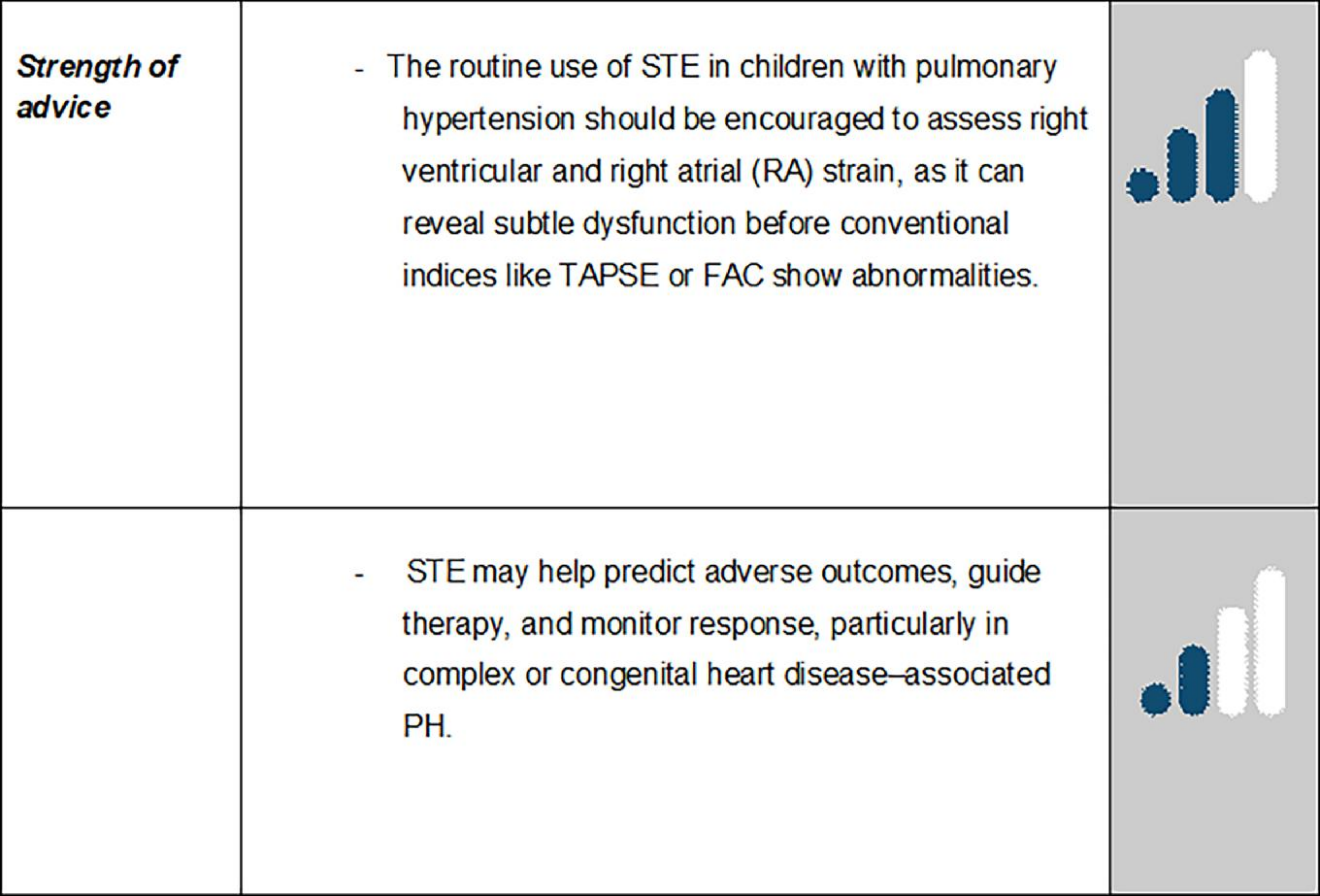
Clinical advice on application of STE in pulmonary hypertension.

**Gaps in knowledge:** (*Figure 14*) The long-term prognostic value, and optimal monitoring intervals for STE-derived atrial and ventricular strain in paediatric pulmonary hypertension remain incompletely defined, limiting widespread implementation in routine care.

#### Systemic hypertension, dialysis, kidney transplant, obesity, and other cardiac risk factors

The early identification of cardiovascular dysfunction in children with chronic diseases or systemic risk factors is crucial, as subtle myocardial changes often precede overt heart failure or clinically detectable remodelling. Conventional echocardiography has limited sensitivity in uncovering subclinical dysfunction, whereas STE, particularly GLS, has proven to be a sensitive tool for detecting early myocardial abnormalities across a wide range of conditions,^213–231^ including systemic hypertension,^216^ chronic kidney disease, dialysis, and kidney transplantation,^217–222^ obesity,^223–226^ autoimmune disorders such as systemic lupus erythematosus,^228–230^ and genetic syndromes or endocrine diseases.^230,231^ In paediatric systemic hypertension, STE demonstrates reduced GLS (−17.8% vs. −20.4%, *P* < 0.001) and circumferential strain abnormalities even with preserved EF, with strain impairment correlating with systolic blood pressure load (*r* = −0.55, *P* < 0.01).^207,211^ Children exposed to hypertensive disorders *in utero* also show early strain impairment (*P* < 0.05).^220^ In children with chronic kidney disease, GLS is consistently reduced compared with controls (*P* < 0.01), providing incremental insight into diastolic dysfunction even when EF is normal.^217^ After kidney transplantation, reductions in GLS (−18.1% vs. −20.3%, *P* < 0.001) correlate with arterial stiffness (*r* = 0.41, *P* < 0.05), and 3D-STE demonstrates involvement of both LV and RV strain (*P* < 0.05).^218,219^ In obesity and cardiometabolic risk, STE detects subclinical systolic dysfunction despite preserved EF, with GLS reductions reported in overweight and obese children (−17.6% vs. −20.2%, *P* < 0.01) and correlations with carotid intima-media thickness (*r* = −0.48, *P* < 0.01).^224^ In paediatric systemic lupus erythematosus, both LV and RV strain are impaired, correlating with disease activity (*P* < 0.05), despite conventional echocardiographic parameters being normal.^227–229^ Finally, STE has revealed early myocardial impairment in other risk conditions, such as 22q11.2 deletion syndrome (*P* < 0.05) and euthyroid Hashimoto’s thyroiditis (*P* < 0.01), highlighting its broad utility in detecting subclinical myocardial involvement across diverse systemic and genetic conditions.^230,231^ Collectively, these studies underscore the role of STE as a sensitive, non-invasive tool for early detection, risk stratification, and longitudinal monitoring of paediatric populations at risk for cardiovascular dysfunction.

##### Clinical advice

**Figure 15:**
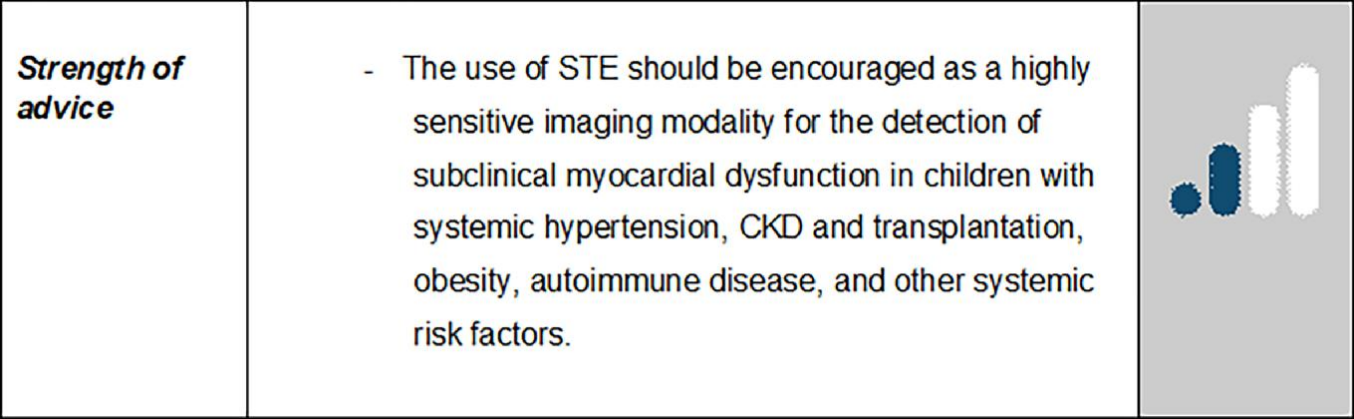
Clinical advice on application of STE in systemic hypertension, dialysis, kidney transplant, obesity, and other cardiac risk factors.

**Gaps in knowledge:** (*Figure 15*) The incorporation of STE into risk assessment protocols for high-risk paediatric populations holds promise. However, multicentre, longitudinal studies are needed to validate thresholds, and determine the prognostic significance of early strain abnormalities.

### Quality improvement

#### Training and teaching

Despite the great potential of STE in paediatric and congenital heart disease, there are still clear gaps in knowledge and practical expertise. Many clinicians are uncertain about the optimal acquisition techniques, how to interpret strain values in children of different ages and sizes, and how to deal with inter-vendor variability. Focused training and teaching programmes are key to overcoming these challenges. Hands-on workshops, interactive online modules, and mentorship opportunities can help clinicians build confidence and consistency in performing and interpreting STE. By embedding these efforts into everyday practice, STE can become a reliable tool for improving the care of children with heart disease.

#### Research and future directions

Research is essential to fill the remaining knowledge gaps and guide the clinical use of STE. Questions remain regarding the prognostic significance of strain measures, the optimal timing for serial assessments, and their application in complex congenital heart defects. Prospective studies, multicentre paediatric registries, and long-term follow-up investigations are needed to provide robust evidence for defining age-specific normal ranges, establishing clinically meaningful cut-offs, and validating emerging software platforms. Collaborative multicentre paediatric registries may facilitate the collection of large-scale datasets representative of diverse patient populations and disease conditions.

Future research should also explore the role of emerging technologies in advancing STE applications. Artificial intelligence-assisted strain analysis and automated contouring algorithms may improve reproducibility, reduce operator dependency, and enhance workflow efficiency. Integration of STE with complementary imaging modalities, particularly CMR feature tracking, may provide multimodality validation and a more comprehensive assessment of myocardial mechanics. The potential additional value of newer STE techniques, including myocardial work and blood speckle tracking,^232,233^ also requires further investigation. Furthermore, machine-learning approaches using large imaging datasets may improve prognostic stratification by identifying complex patterns associated with outcomes and disease progression.

Linking research directly to training ensures that clinicians remain continuously updated with evolving technologies and scientific evidence, creating a cycle in which evidence informs clinical practice and clinical practice, in turn, generates new research questions.

## Conclusions and limitations

**Figure 16:**
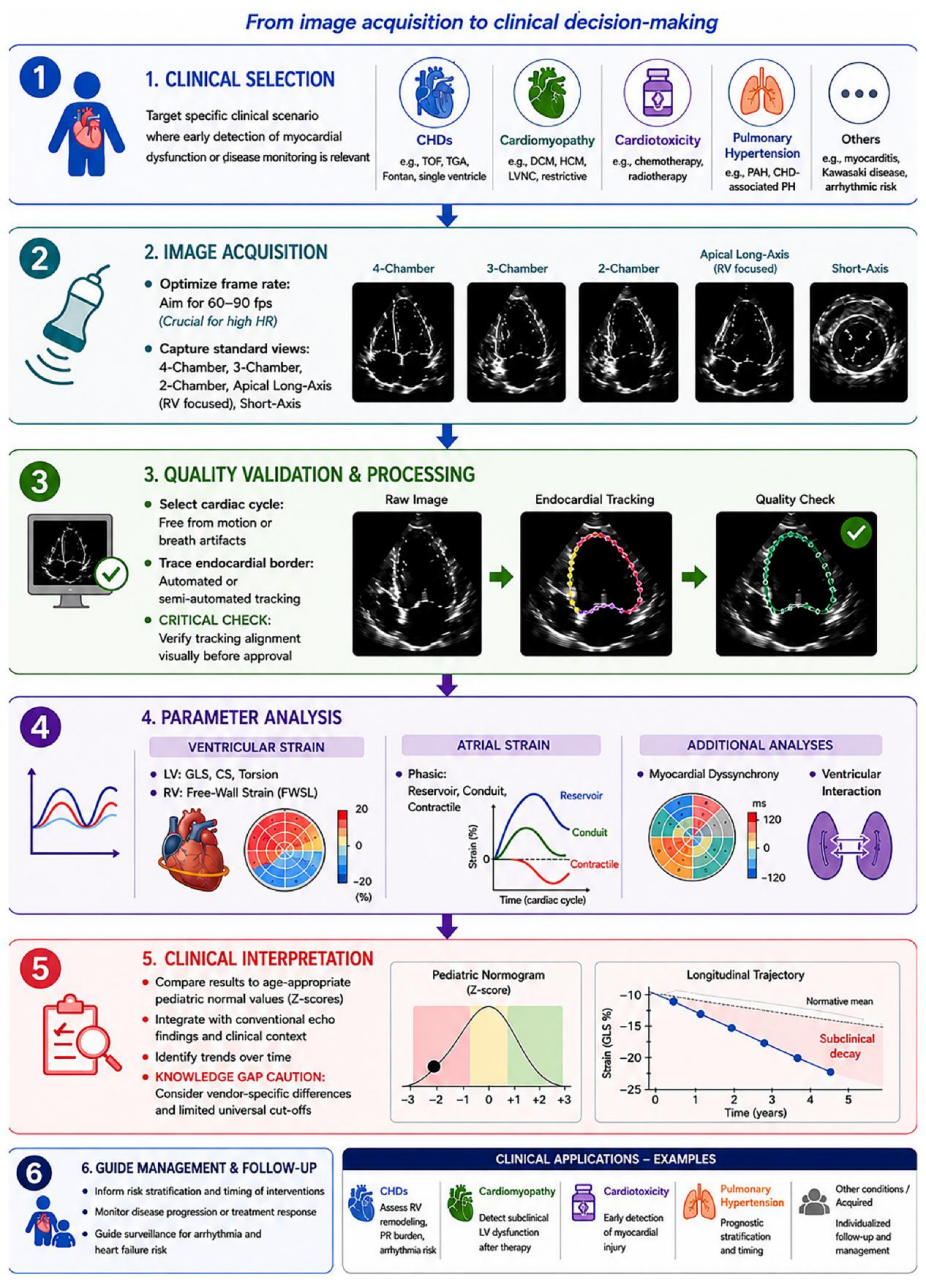
Practical integration of STE in paediatric and congenital echocardiography: from imaging to decision making.

**Figure 17:**
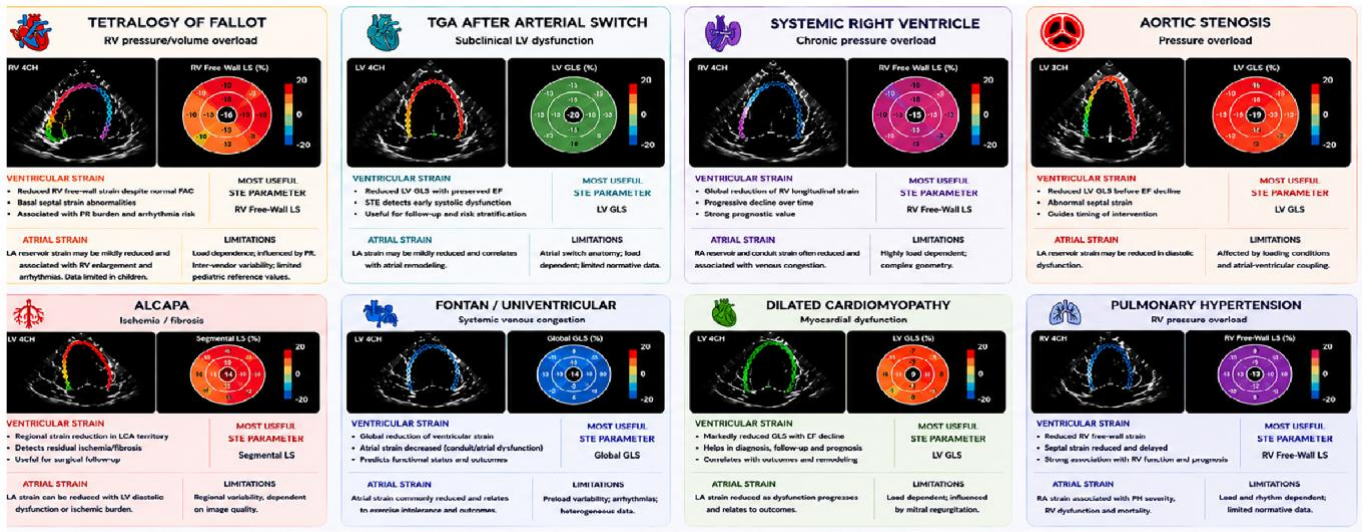
Examples of main applications of STE in paediatric and congenital echocardiography.

This consensus document provides an overview and practical guidance for the use of STE in paediatric and congenital heart disease (*Figures 16 and 17*). We cover application of STE in a broad range of congenital heart defects and acquired cardiac defects due to multiple diseases (e.g. rhythm disturbances, cardiomyopathy, cardiotoxicity, pulmonary hypertension, inflammatory disease, systemic hypertension, kidney diseases, obesity, etc.) offering details on advantages and limitations.

Limitations to the systematic use of STE were also elucidated including inter-vendor variability, acoustic window quality, level of children cooperation, the influence of loading conditions and heart rate on strain measurements, and the challenges in interpreting STE in complex congenital anatomies or in very young patients. The document further emphasizes the current lack of data on the clinical and prognostic value of STE, underlining the need for multicentre studies, standardized protocols, and larger datasets to validate and reinforce the existing evidence.

## Data Availability

No new data were generated or analysed in support of this research.
